# The *Burkholderia bcpAIOB* Genes Define Unique Classes of Two-Partner Secretion and Contact Dependent Growth Inhibition Systems

**DOI:** 10.1371/journal.pgen.1002877

**Published:** 2012-08-09

**Authors:** Melissa S. Anderson, Erin C. Garcia, Peggy A. Cotter

**Affiliations:** Department of Microbiology and Immunology, School of Medicine, The University of North Carolina at Chapel Hill, Chapel Hill, North Carolina, United States of America; University of Toronto, Canada

## Abstract

Microbes have evolved many strategies to adapt to changes in environmental conditions and population structures, including cooperation and competition. One apparently competitive mechanism is contact dependent growth inhibition (CDI). Identified in *Escherichia coli*, CDI is mediated by Two–Partner Secretion (TPS) pathway proteins, CdiA and CdiB. Upon cell contact, the toxic C-terminus of the TpsA family member CdiA, called the CdiA-CT, inhibits the growth of CDI^−^ bacteria. CDI^+^ bacteria are protected from autoinhibition by an immunity protein, CdiI. Bioinformatic analyses indicate that CDI systems are widespread amongst α, β, and γ proteobacteria and that the CdiA-CTs and CdiI proteins are highly variable. CdiI proteins protect against CDI in an allele-specific manner. Here we identify predicted CDI system-encoding loci in species of *Burkholderia*, *Ralstonia* and *Cupriavidus*, named *bcpAIOB*, that are distinguished from previously-described CDI systems by gene order and the presence of a small ORF, *bcpO*, located 5′ to the gene encoding the TpsB family member. A requirement for *bcpO* in function of BcpA (the TpsA family member) was demonstrated, indicating that *bcpAIOB* define a novel class of TPS system. Using fluorescence microscopy and flow cytometry, we show that these genes are expressed in a probabilistic manner during culture of *Burkholderia thailandensis* in liquid medium. The *bcpAIOB* genes and extracellular DNA were required for autoaggregation and adherence to an abiotic surface, suggesting that CDI is required for biofilm formation, an activity not previously attributed to CDI. By contrast to what has been observed in *E. coli*, the *B. thailandensis bcpAIOB* genes only mediated interbacterial competition on a solid surface. Competition occurred in a defined spatiotemporal manner and was abrogated by allele-specific immunity. Our data indicate that the *bcpAIOB* genes encode distinct classes of CDI and TPS systems that appear to function in sociomicrobiological community development.

## Author Summary

Contact dependent growth inhibition (CDI) is a phenomenon discovered in *Escherichia coli* in which CDI^+^ bacteria inhibit the growth of CDI^−^ bacteria upon cell-to-cell contact. CDI is mediated by large toxic “exoproteins” present on the bacterial cell surface. An ‘immunity’ protein protects CDI^+^ cells from killing themselves. While predicted CDI systems are widespread throughout bacterial genera, the role of these systems in nature has remained elusive. Here we identify a distinct class of CDI system in *Burkholderia* species. The genes encoding these systems are expressed in a stochastic manner such that only a few cells in the population produce the proteins at any given time when grown in broth. We also show that these systems are required for aggregation on an abiotic surface, suggesting an important role for CDI in biofilm formation. Finally, we show that CDI mediates competition under specific conditions in a precise spatiotemporal pattern when bacteria are grown on a solid surface. Our data suggest that in nature, CDI systems may be used by bacteria to establish complex sociomicrobial communities.

## Introduction

Microbes rarely live alone. Whether free in the environment or in close association with eukaryotic hosts, microbes typically share their living space with other viral, prokaryotic, and/or eukaryotic microorganisms. Survival under these conditions requires mechanisms for sensing, responding to, and cooperating or competing with neighboring organisms. Contact dependent growth inhibition (CDI) systems are protein toxin delivery mechanisms that appear to be involved in interbacterial competition [1]. CDI was discovered in *Escherichia coli* strain EC93 due to its ability to inhibit the growth of specific CDI^−^ *E. coli* strains upon cell-to-cell contact. CDI is mediated by Two–Partner Secretion (TPS) system proteins CdiA and CdiB [1]. TPS systems are widespread amongst Gram-negative bacteria. They export large exoproteins (TpsA family members such as CdiA) across the outer membrane using pore-forming β-barrel proteins (TpsB family members such as CdiB) [2], [3]. Functions attributed to TpsA proteins before the discovery of CDI included adherence to eukaryotic cells, induction of cytolysis in host cells, iron uptake, and autoaggregation [2], [3]. Characterization of CDI in *E. coli* revealed an additional TpsA-mediated function: inhibition of ‘target’ bacterial cell growth upon contact. CDI^+^ bacteria are protected from autoinhibition because they produce CdiI, a 79 amino acid ‘immunity’ protein encoded immediately 3′ to *cdiA* [1].

Research in our lab on *Burkholderia pseudomallei* led to the discoveries that genes predicted to encode CDI systems are present in a large number of α-, β-, and γ-proteobacteria, that the C-terminal ∼300 aa of CdiA proteins (CdiA-CTs) and CdiI proteins are highly variable, and that CdiA-CTs are sufficient to confer toxicity when produced intracellularly in *E. coli* [4]. Some CdiA-CTs have been demonstrated to possess nuclease activity, functioning as DNases or tRNases [4], [5]. CdiI proteins bind to cognate CdiA-CT proteins (those encoded by the same *cdi* locus), blocking their nuclease activity, but not heterologous CdiA-CT proteins (those encoded by different *cdi* loci) [4], [5]. Experiments with *E. coli* strains producing chimeric CdiA proteins showed that CdiI proteins provide immunity against interbacterial growth inhibition in an allele-specific manner, conferring protection only towards cognate CdiA-CTs but not heterologous CdiA-CTs [4]. Although chimeric CdiA proteins containing CdiA-CTs encoded by different species of bacteria were effective at mediating interbacterial competition in *E. coli*, CDI has so far only been shown to function between members of the same species [1], [4]. The current model for CDI states that, upon cell-to-cell contact with a closely related bacterium (possibly by interacting directly with the outer membrane protein BamA [6]), the CdiA-CT from a CDI^+^ cell is delivered to the cytoplasm of the target cell where it inhibits cell growth by degrading DNA or specific tRNAs. If present in the target cell, the cognate CdiI immunity protein binds to the CdiA-CT, blocking its nuclease activity [4]. While considerable insight has been gained regarding how CDI systems function mechanistically in *E. coli*, almost nothing is known about when or why these systems are deployed by bacteria in nature.

Initial bioinformatic analysis of available bacterial genomes revealed that putative CDI systems fall into two distinct classes, “*E. coli*-type,” which include systems found in bacterial genera other than *Burkholderia*, and “*Burkholderia*-type,” found only in *Burkholderia* spp [4]. *E. coli*-type CDI systems are encoded by genes with the order *cdiBAI* and predicted CdiA proteins contain a highly conserved VENN motif that separates the conserved (∼2700 aa) N-terminus from the variable (∼300 aa) C-terminus (the CdiA-CT). *Burkholderia*-type CDI systems are encoded by genes with the order *cdiAIB* and putative CdiA proteins contain an NxxLYN motif instead of VENN [4]. Whether the *Burkholderia* proteins actually function as CDI systems has not yet been demonstrated.

*Burkholderia* spp are Gram-negative soil saprophytes and many are opportunistic pathogens [7], [8], [9]. *Burkholderia cepacia* complex (Bcc) strains, for example, cause life-threatening respiratory infections in cystic fibrosis patients [10], [11], and *B. pseudomallei* strains cause melioidosis, a disease that can range from localized wound infections and abscesses to fulminant pneumonia and septicemia [9], [12]. Because it is highly virulent by the aerosol route, resistant to most commonly used antibiotics, and extremely closely related to *Burkholderia mallei*, which has been used as a bioterrorism agent in the past, *B. pseudomallei* is an NIAID Category B Biothreat pathogen and select agent [13]. Working with *B. pseudomallei* in the laboratory requires BSL-3 practices and rigorous security measures. *Burkholderia thailandensis* is closely related to *B. pseudomallei* and occupies the same environmental niche (both are endemic to southeast Asia and northern Australia) [14], [15], [16], but is not a human pathogen, is not a select agent, and requires only BSL-1 practices.

Here, we characterize the unique class of CDI systems produced by *Burkholderia* spp. We show that these systems compose a novel class of TPS system that requires a third protein for the large exoprotein to function, that expression of *Burkholderia* CDI protein-encoding genes is regulated in a probabilistic manner, that the gene products contribute to biofilm formation, and that CDI-mediated interbacterial competition in *Burkholderia* occurs on solid surfaces in a unique temporal and spatial pattern.

## Results

### *bcpAIOB* genes of *Burkholderia* spp compose a unique class of CDI system-encoding loci

#### Identification and general features of *Burkholderia*-type CDI system-encoding loci

**Figure 1:**
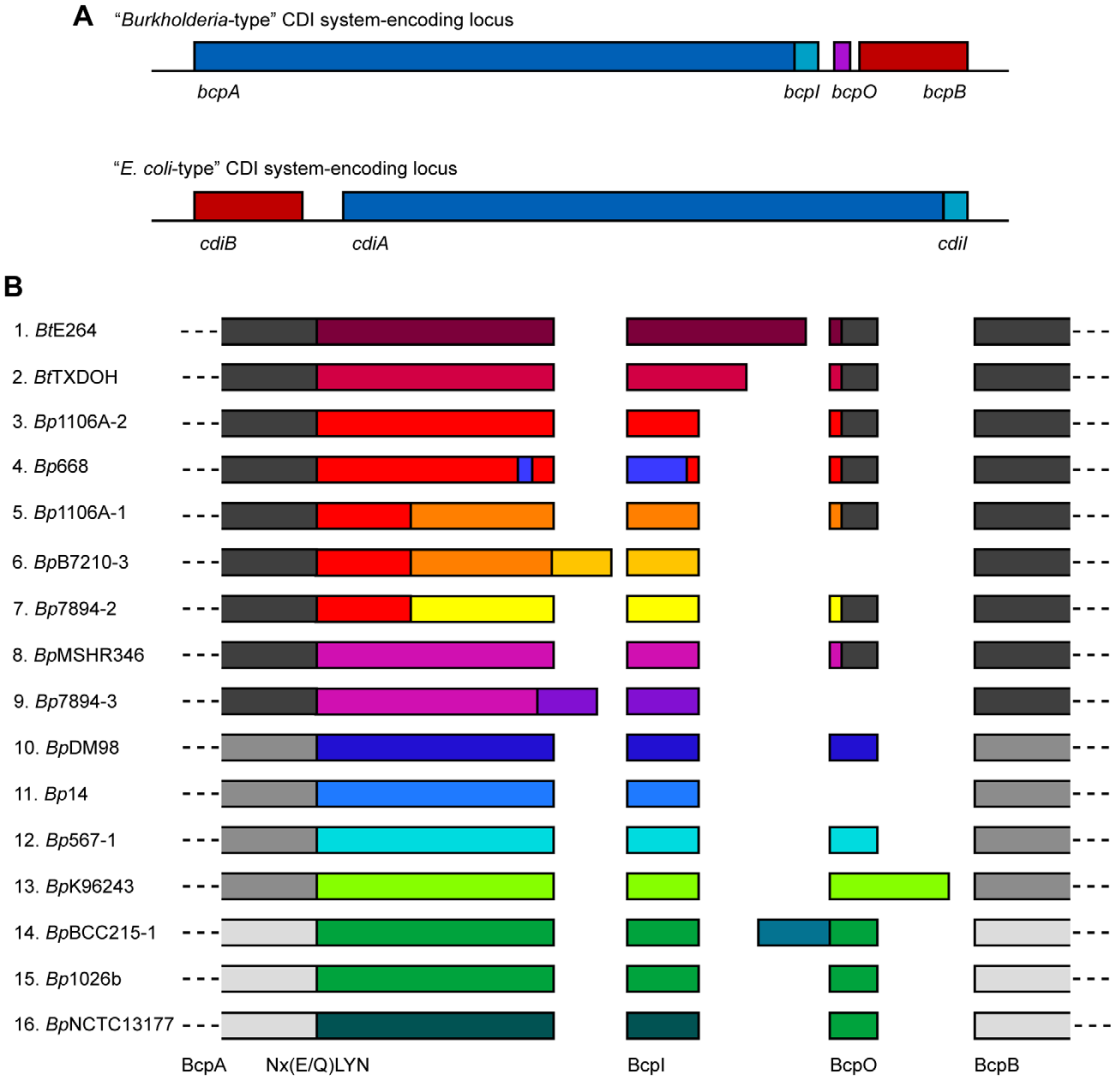
Organization and diversity of *Burkholderia*-type CDI-encoding loci. A) Schematic of *Burkholderia*- and *E. coli*-type CDI-encoding loci. *Burkholderia*-type CDI loci are encoded by genes *bcpAIOB* and differ from *E. coli*-type CDI loci in gene order and content; *Burkholderia*-type CDI loci contain an additional ORF, *bcpO*, not present in *E. coli*-type CDI loci. B) Schematic of the amino acid sequence of a single representative allele for each of the *B. thailandensis* and *B. pseudomallei* CDI-encoding loci. The alleles have strong sequence similarity (∼89% similar) amongst the N-terminal encoded ∼2750 aa of BcpA, BcpB, and some BcpO proteins, but fall into three phylogenetic groups (indicated by shades of gray). The diversity of the CDI-encoding proteins is located within the ∼350 aa C-terminal to the Nx(E/Q)LYN motif in BcpA, BcpI, and some BcpO proteins, with no more than ∼10% similarity between any two alleles (indicated by different colors). For some BcpO proteins, the diversity is restricted to the predicted signal sequence peptide (alleles 1–5, 7, and 8) whereas the functional domain of the protein is highly conserved.

Initial comparisons of putative CDI system-encoding loci included six from *Burkholderia* spp and indicated that the *Burkholderia* loci had a different gene order and a different motif separating the conserved and variable regions of the predicted CdiA orthologs compared with *E. coli*-type CDI system-encoding loci [4]. Upon closer inspection of putative *cdi* loci in *B. pseudomallei* strains, we noticed an additional small open reading frame (ORF) located between the predicted *cdiI* and *cdiB* homologs (Figure 1A). Because further investigation indicated that *Burkholderia*-type CDI systems form a distinct class of CDI system and also a novel class of TPS system, we chose to give them a distinct nomenclature. We therefore named the genes encoding *Burkholderia*-type CDI systems *bcpAIOB*, for *Burkholderia* CDI proteins A, I, O and B.

To identify all *Burkholderia*-type CDI-encoding loci in organisms with available genome sequence information, we performed a BLAST search using the predicted BcpB protein from *B. pseudomallei* K96243, downloaded approximately 20 kbp of DNA sequence flanking each identified *bcpB* homolog, and used Vector NTI to identify ORFs and to perform sequence comparisons. We found 58 loci in which there was a large ORF (∼10 kb) predicted to encode a TpsA protein (the *bcpA* homolog) 5′ to the *bcpB* homolog and a small ORF (∼300 bp) immediately 3′ to the *bcpA* homolog (Table S1, Figure S1). (Note that finished genome sequences were available for four strains of *B. pseudomallei* and about half of the other organisms in which we identified *Burkholderia*-type CDI-encoding loci (as indicated in Table S1). The other loci were identified in publically available contigs from unfinished genome sequencing projects.) Thirty of the identified loci were present in 20 *B. pseudomallei* strains (four strains contain two loci and three strains contain three loci) and two were identified in *B. thailandensis* strains. The other 26 loci were present in Bcc strains (*Burkholderia ambifaria*, *Burkholderia cenocepacia*, *Burkholderia dolosa*, *Burkholderia multivorans*, and *Burkholderia vietnamiensis*), *Burkholderia gladioli*, *Burkholderia glumae*, *Burkholderia phymatum*, *Burkholderia rhizoxinica*, *Burkholderia ubonensis*, *Burkholderia xenovorans*, *Ralstonia solanacearum*, *Ralstonia syzygii*, and *Cupriavidus metallidurans*. *Ralstonia* and *Cupriavidus* are the two genera most closely related to *Burkholderia*. No *Burkholderia*-type CDI-encoding loci were identified in any other bacteria. However, *E. coli*-type CDI-encoding loci were identified in some closely related bacteria (e.g., *Cupriavidus taiwanensis*) [4]. In no case were both *E. coli*-type and *Burkholderia*-type CDI-encoding loci present in a single strain or even different strains of the same species.

Amongst all identified *Burkholderia*-type CDI-encoding loci, predicted BcpA proteins ranged in size from 2814 aa to 3651 aa, with most about 3100 aa, and nearly all contained a Nx(E/Q)LYN motif located approximately 350 aa from the C-terminus. Predicted BcpI proteins ranged in size from 44 aa to 301 aa, with most about 100 aa. In all strains except one, the first codons of the *bcpI* genes were within 11 bp of the stop codons of the *bcpA* genes, and in some cases they were 5′ to the *bcpA* stop codons, suggesting that the *bcpA* and *bcpI* genes may be both transcriptionally and translationally coupled. The one strain that differed in this respect was *B. thailandensis* TXDOH in which the predicted *bcpI* gene was located 313 bp 3′ to the end of the *bcpA* gene. The predicted BcpA-CT of this strain (i.e., the region of the BcpA protein from the Nx(E/Q)LYN motif to the C-terminus) is about 90 aa shorter than most of the other BcpA-CTs, suggesting the possibility that the identified *bcpA* stop codon is the result of a sequencing error. However, translating the DNA sequence between the identified stop codon and the 5′ end of the identified *bcpI* gene in all three reading frames did not result in one continuous ORF without stop codons, so the discrepancy with this strain cannot be explained by a single nucleotide sequencing error. In all but eight loci, a small ORF was present between the *bcpI* and *bcpB* genes. We named this ORF *bcpO*.

#### Comparison of *B. pseudomallei* and *B. thailandensis bcpAIOB* genes

The 32 loci present in *B. pseudomallei* and *B. thailandensis* strains comprised 16 different alleles in which the nucleotide sequences from 5′ of the *bcpA* gene to 3′ of the *bcpB* gene (i.e., the entire *bcpAIOB* locus) were nearly identical (Table 1, Table S1). Some alleles were present in multiple strains (e.g., each of the three different alleles present in *B. pseudomallei* 1106A is present in other strains), while others are present in only a single strain. If a strain contains more than one allele, the alleles are different (duplicated alleles in the same strain were not found). Moreover, Tuanyok *et al.* identified genomic islands in five *B. pseudomallei* strains and found that these loci (which they referred to as ‘*fha* gene clusters’) were present on genomic islands [17]. Based on the limited number of strains used in that study, it appears that specific alleles are associated with specific genomic islands, i.e., if two strains contain the same allele, the alleles are present in the same location (same genomic island) in the chromosome.

**Table 1:** Predicted *B. thailandensis* and *B. pseudomallei* CDI-system encoding alleles.

No.	Allele(s)	BcpA^3^	BcpI^3^	BcpO^3^	Signal Peptide^4^
1	1 *Bt*E264*	3147	270	74	Yes (20–21)^5^
2	*Bt*TXDOH	3025	171	73	Yes (19–20)
3	2 *Bp*1106A-2*, *Bp*B7210-2, *Bp*1710b-1*, *Bp*112-2, *Bp*Pasteur52237	3131	117	74	Yes (20–21)
4	*Bp*668*	3134	118	74	Yes (20–21)
5	*Bp*1106A-1*, *Bp*BCC215-2	3123	99	73	Yes (19–20)
6	*Bp*B7210-3	3223	97	–	–
7	*Bp*7894-2	3125	88	73	Yes (28–29)
8	*Bp*MSHR346, *Bp*305, *Bp*406e, *Bp*7894-1, *Bp*576-2	3107	96	74	Yes (20–21)
9	*Bp*7894-3	2980	83	–	–
10	*Bp*DM98, *Bp*S13, *Bp*1710b-2*, *Bp*1655, *Bp*B7210-1, *Bp*112-1, *Bp*1106A-3*	3141	109	57	No
11	*Bp*14	3057	166	–	–
12	*Bp*576-1	3083	99	81	No
13	*Bp*K96243*, *Bp*91	3103	109	172	No
14	*Bp*BCC215-1	3122	101	147	No
15	*Bp*1026b	3122	101	81	No
16	*Bp*NCTC13177	3096	111	81	No

1 *Bt*, *Burkholderia thailandensis*;

2 *Bp*, *Burkholderia pseudomallei*.

3 Amino acid length of predicted protein.

4 Signal peptide in BcpO;

5 Amino acid position of predicted cleavage site.

* Loci from complete genome sequences.

The predicted aa sequences of the BcpB proteins encoded by all alleles were highly similar (89% similarity with the consensus sequence), but separated into three groups phylogenetically (Figure 1B). Similarly, the predicted aa sequences of the N-terminal ∼2750 aa of the BcpA proteins encoded by all alleles (N-terminal to the Nx(E/Q)LYN motif) were highly similar, but also separated into the same three phylogenetic groups. In contrast, the aa sequences of the C-terminal ∼350 aa of the BcpA proteins encoded by each of the different alleles were typically less than 10% similar. For a few of the alleles, however, the BcpA proteins appeared to be mosaics, with only the last ∼100–200 aa being different (e.g., alleles 3–7, and alleles 8 and 9 in Figure 1B). In all cases, the predicted aa sequences of the BcpI proteins differed dramatically with no more than 10% similarity between any two alleles. In a few of the *E. coli*-type CDI systems, the immunity proteins have been shown to bind to cognate CdiA-CTs in an allele-specific manner [4]. If the *Burkholderia*-type CDI systems function analogously and the immunity proteins for alleles 3–9 function in an allele-specific manner, it will suggest that BcpI binds to the C-terminal ∼100–200 aa of BcpA.

#### Bioinformatic analysis of the small ORFs (*bcpO* genes) unique to *Burkholderia*-type CDI-encoding loci

The predicted BcpO proteins encoded by alleles 1–5, 7, and 8 are 73 or 74 aa long and contain N-terminal signal sequences with lipoboxes. Although the signal sequences of the preproteins encoded by the different alleles vary, the predicted mature proteins are nearly identical, i.e., the variation is located within the signal sequence. None of the predicted BcpO proteins contains a Lol avoidance signal (an Asp residue following the N-terminal Cys of the mature lipoprotein), therefore these BcpO lipoproteins are predicted to localize to the inner leaflet of the outer membrane. The fact that the mature BcpO proteins are identical within this family suggests that they play a conserved, non-allele-specific function, however they share no similarity to other characterized proteins.

The predicted BcpO proteins for alleles 10–16 are more heterogeneous, and their sequences vary in an allele-specific manner with their cognate BcpA-CT and BcpI proteins for the most part (alleles 14–16 being exceptions). They vary in size from 57 aa to 172 aa. None contains a predicted N-terminal signal sequence, and they have no similarity to any characterized proteins or protein domains.

### The *bcpAIOB* genes are transcribed as an operon in *B. thailandensis*

**Figure 2:**
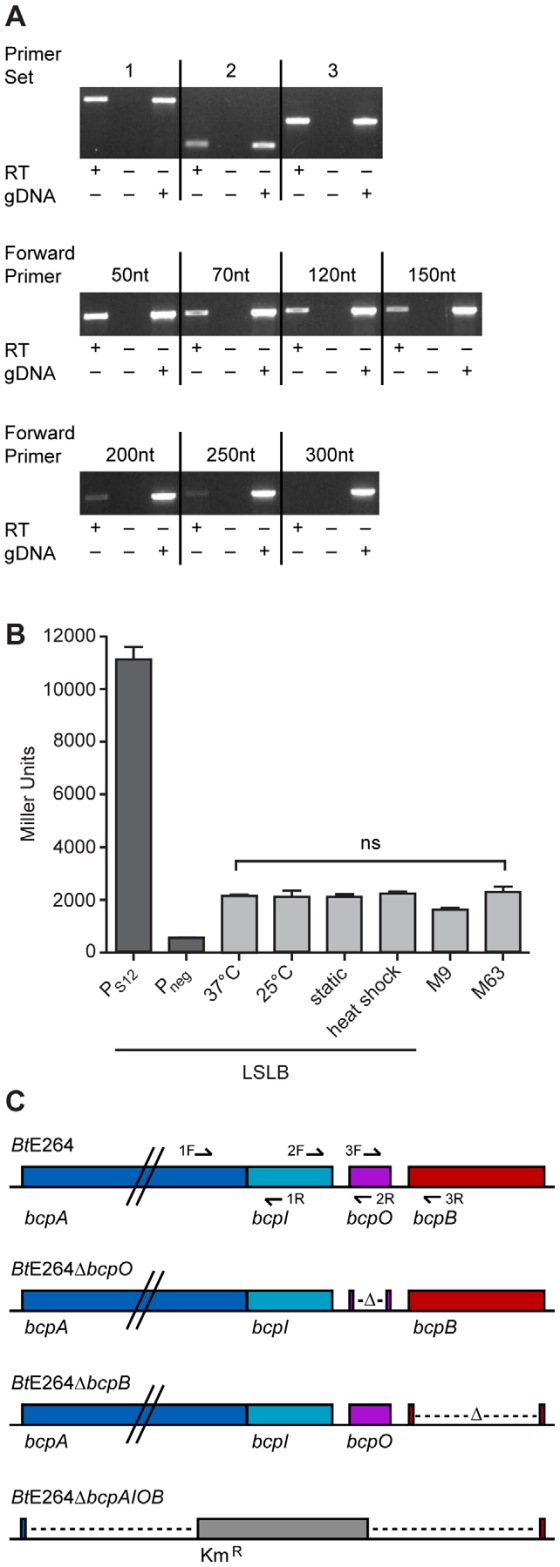
Examination of the *bcpAIOB* locus in *B. thailandensis*. A) Top panel, analysis of the operon structure of *bcpAIOB*. Primer sets 1, 2, and 3 flanking the intergenic region of *bcpA* and *bcpI*, *bcpI* and *bcpO*, and *bcpO* and *bcpB*, respectively, (as indicated in 2C) were used for RT-PCR. Middle and bottom panels, analysis of the approximate start site of transcription of *bcpA*. RT-PCR was performed using primers annealing 50, 70, 120, 150, 200, 250, and 300 nt 5′ to *bcpA* with a reverse primer internal to *bcpA*. All products were visualized by ethidium bromide. B) Expression of *bcpA*. P_S12_-*lacZ* and P_neg_-*lacZ* (dark gray bars) and P*_bcpA_*-*lacZ* (light gray bars) reporter strains were cultured under various conditions and assayed for β-galactosidase activity. Error bars represent the mean ± 1 SEM. C) Schematic of *B. thailandensis* E264 strain constructs, including WT, Δ*bcpO*, Δ*bcpB*, and Δ*bcpAIOB*, used throughout the study.

To investigate the operon structure of the *bcp* locus in *B. thailandensis* strain E264, reverse transcriptase (RT) PCR was performed on RNA extracted from bacteria cultured in low salt LB broth (LSLB), the standard medium used for culturing *B. thailandensis*. Primer sets (Table S3) flanking the junctions between *bcpA* and *bcpI*, *bcpI* and *bcpO*, and *bcpO* and *bcpB* (1, 2, and 3, respectively, in Figure 2C) yielded products of the expected sizes (Figure 2A, top panel), indicating the *bcpAIOB* genes form an operon. RT-PCR was performed to identify the approximate transcription start site using forward primers annealing 5′ to *bcpA* with an internal *bcpA* reverse primer. Products of the expected size were obtained for forward primers annealing 50, 70, 120, and 150 nt 5′ to *bcpA* (Figure 2A, middle panel), indicating the transcription start site for *bcpA* is at least 150 nt 5′ of the translation start site. Faint products were obtained for forward primers annealing 200 and 250 nt 5′ to *bcpA* (Figure 2A, bottom panel), but no product was obtained with the forward primer annealing 300 nt 5′ to *bcpA*, suggesting the possibility of a second promoter located 250–300 nt 5′ to the translation start site.

To measure expression of *bcpAIOB*, we constructed a strain containing a *bcpA* promoter-*lacZ* fusion (P*_bcpA_*-*lacZ*) inserted at the *att*Tn7 site on the chromosome in *B. thailandensis* E264 and measured β-galactosidase activity in cells cultured under various conditions. For comparison, we also constructed two additional strains, one in which the promoter of the gene encoding the ribosomal S12 subunit (P_S12_) was fused to *lacZ* and one with no promoter (P_neg_) 5′ to *lacZ*. Approximately 2,000 Miller units of β-galactosidase activity were produced in the P*_bcpA_*-*lacZ* fusion strain cultured under all conditions tested (Figure 2B). The P_S12_-*lacZ* fusion produced ∼11,000 Miller units of β-galactosidase activity and the P_neg_-*lacZ* fusion produced ∼500 Miller units of β-galactosidase activity in cells cultured in LSLB broth. These data suggest *bcpA* is transcribed at relatively low levels under each of the culture conditions tested.

We next constructed strains with in-frame deletion mutations in genes in the *bcpAIOB* operon. While it was possible to construct a Δ*bcpO* strain and a Δ*bcpB* strain by allelic exchange, and a strain in which the entire *bcpAIOB* operon was replaced with a gene encoding kanamycin resistance by natural transformation (Figure 2C), it was not possible to construct a Δ*bcpI* strain, suggesting *bcpI* is essential or, analogous to the *E. coli* CDI system, BcpI is required to protect against BcpA-mediated toxicity. For complementation experiments, we constructed plasmids to deliver *bcpO* or *bcpB*, driven by the constitutively active P_S12_ promoter or the native promoter (P*_bcpA_*) to the *att*Tn7 site. All mutant strains grew equally compared to wild type E264 in LSLB medium (data not shown).

### BcpA protein production in wild type, Δ*bcpO*, and Δ*bcpB B. thailandensis*

**Figure 3:**
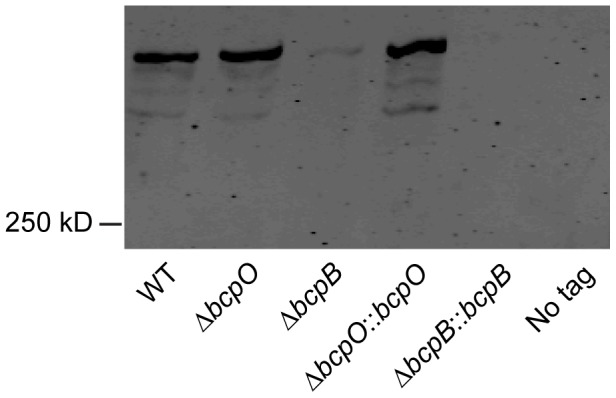
Analysis of BcpA-HA. Immunoblot of whole cell lysates of WT (E264BcpA-HA::pECG22), Δ*bcpO* (E264BcpA-HAΔ*bcpO*::pECG22), Δ*bcpB* (E264BcpA-HAΔ*bcpB*::pECG22), Δ*bcpO*::*bcpO* (E264BcpA-HAΔ*bcpO*::*bcpO*::pECG22), Δ*bcpB*::*bcpB* (E264BcpA-HAΔ*bcpB*::*bcpB*::pECG22), no tag (E264::pECG22) strains producing BcpA-HA were separated by SDS-PAGE and stained with anti-HA antibody.

Initial attempts to investigate the contribution of *bcpO* and *bcpB* to BcpA production were carried out by performing Western blots from cell lysates of bacteria expressing the *bcpAIOB* genes from their native promoter, P*_bcpA_*, and producing BcpA containing a hemagglutinin (HA) epitope (BcpA-HA) N-terminal to the predicted Nx(E/Q)LYN sequence of the mature protein (i.e., immediately C-terminal to F2633, 141 amino acids from the N-terminal side of the Nx(E/Q)LYN sequence). We were unable to detect BcpA in this strain, possibly because *bcpAIOB* expression was insufficient under the growth conditions used. We therefore constructed *B. thailandensis* strains in which the *bcpAIOB* operon was controlled by the constitutively active ribosomal S12 subunit promoter, P_S12_. In immunoblots of whole cell lysates of otherwise wild type *B. thailandensis* (E264BcpA-HA::pECG22), anti-HA antibodies recognized a polypeptide with considerably slower mobility than the 250 kDa molecular weight marker (possibly corresponding to 306 kDa, the predicted molecular mass of BcpA), plus three slightly smaller and much less abundant polypeptides (Figure 3). The polypeptide profile detected in the Δ*bcpO* strain was identical to that of E264BcpA-HA::pECG22. By contrast, only a very low level of the largest polypeptide was detected in whole cell lysates of the Δ*bcpB* strain (E264BcpA-HAΔ*bcpB*::pECG22) (Figure 3). In other TPS systems, the TpsA protein is undetectable when the strain contains a loss-of-function mutation in the TpsB-encoding gene, presumably because the TpsA protein, which cannot be translocated across the outer membrane, is degraded in the periplasm [18], [19]. Our data suggest that the same is true for *bcpA* and *bcpB*. Complementation of the Δ*bcpO* and Δ*bcpB* strains with *bcpO* and *bcpB*, respectively, expressed from the P_S12_ promoter did not alter the polypeptide profiles of these strains (Figure 3). However, the fact that BcpA-HA protein was detectable in the Δ*bcpO* strain and not in the Δ*bcpB* strain indicates that the Δ*bcpO* mutation does not have polar effects that abrogate expression of *bcpB*. Similarly, RT-PCR indicated that *bcpB* transcription was not abrogated by the in-frame deletion in the Δ*bcpO* strain (Figure S2).

### The *bcpAIOB* operon is expressed in a probabilistic manner when *B. thailandensis* is cultured in liquid medium

**Figure 4:**
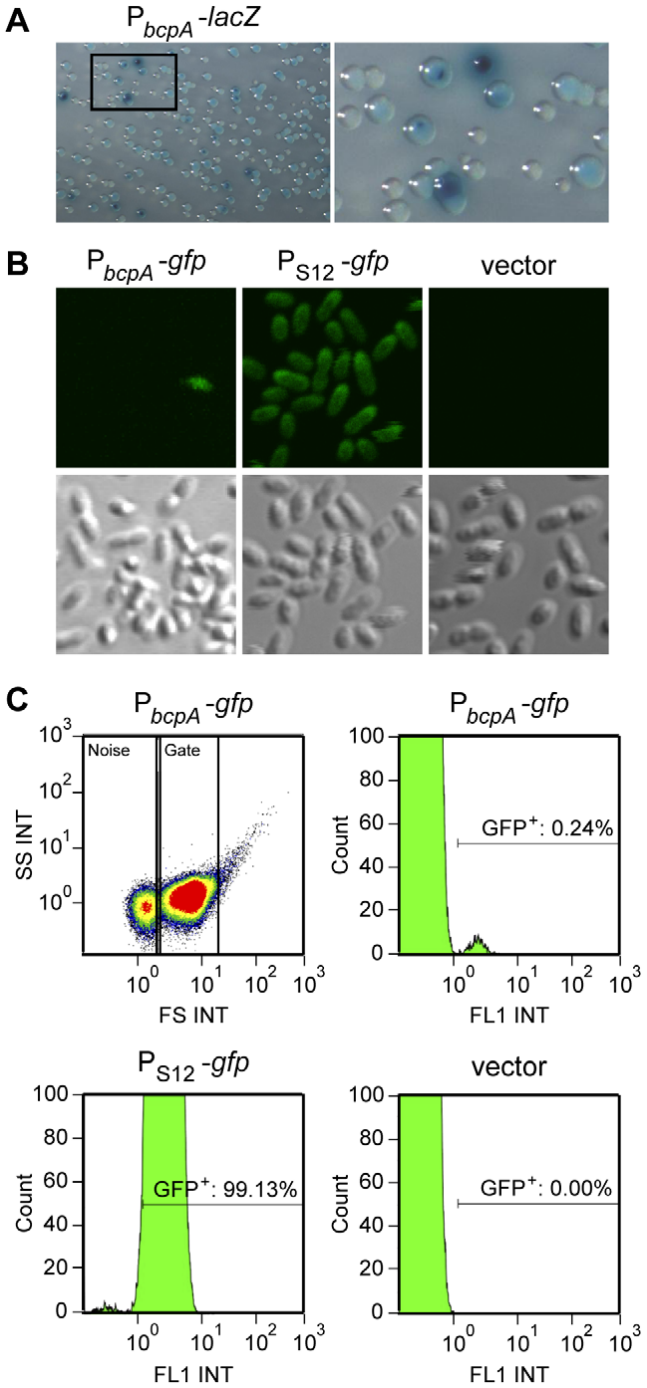
Per cell analysis of the probabilistic expression of *bcpA*. A) P*_bcpA_*-*lacZ* reporter strain plated on M63 minimal medium agar supplemented with X-gal. B) Confocal microscopy of *gfp* reporter strains, P*_bcpA_*-*gfp*, P_S12_-*gfp*, and E264Km^R^ (vector), cultured overnight in liquid medium. C) Representative flow cytometry analysis of *gfp* reporter strains, P*_bcpA_*-*gfp*, P_S12_-*gfp*, and E264Km^R^ (vector), cultured overnight in liquid medium. The dot plot (top left panel) of side scatter intensity (SS INT) vs. forward scatter intensity (FS INT) shows the events corresponding to *B. thailandensis* that were gated on (“Gate”) for subsequent analysis. Histograms show the number of GFP-positive events (Count) vs. relative fluorescence intensity (FL1 INT) for each reporter strain, and the percent of GFP-positive bacteria detected in each culture is indicated.

Upon plating the P*_bcpA_*-*lacZ* fusion strain on solid medium containing X-gal, we found that the colonies were not all the same intensity blue; approximately 5–10% of the colonies were dark blue and 90–95% of the colonies were light blue (Figure 4A). To explore the possibility that *bcpA* was highly expressed in just a small proportion of cells in the population, we constructed fluorescent reporter fusion strains by delivering a *gfp* gene fused to P*_bcpA_* or P_S12_ to the *att*Tn7 chromosomal insertion site in *B. thailandensis* E264. Bacteria were cultured in liquid broth and visualized by confocal microscopy. All bacteria containing the P_S12_-*gfp* fusion produced high levels of GFP (Figure 4B). By contrast, only a few fluorescent bacteria were present in cultures containing the P*_bcpA_*-*gfp* fusion. These data suggest *bcpA* is differentially expressed within the bacterial population.

We next performed flow cytometry to measure *bcpA*-*gfp* expression in a large number of bacterial cells when cultured in liquid medium. Events distinct from the PBS control (Figure S3) were identified as bacteria and gated on for subsequent analysis. Approximately 99% of the bacteria in cultures of the P_S12_-*gfp* fusion strain were GFP^+^ (Figure 4C, Table S2), whereas only ∼0.2% of the P*_bcpA_*-*gfp* fusion containing bacteria were GFP^+^, indicating that *bcpA* is differentially expressed within a population of bacteria when cultured in liquid medium. The mean relative fluorescence intensity of the P*_bcpA_*-*gfp* GFP^+^ cells was similar to that of P_S12_-*gfp* GFP^+^ cells (Table S2), indicating expression was very high in the GFP^+^ P*_bcpA_*-*gfp* bacteria. Taken together, these data show that only a small percentage of bacteria express *bcpA* when cultured in liquid medium, but those that do express *bcpA* do so at a high level.

### The *bcpAIOB* genes are required for autoaggregation in M63 minimal medium

**Figure 5:**
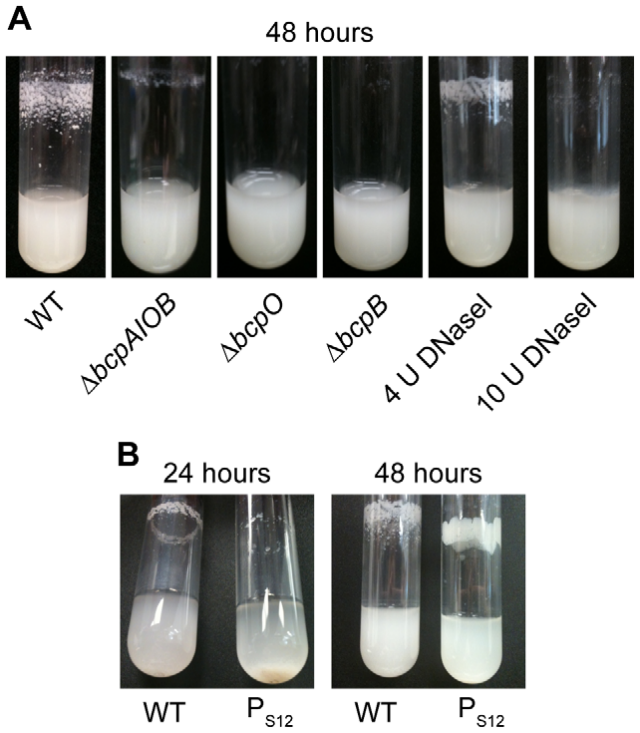
*bcpAIOB*-dependent autoaggregation in M63 minimal medium. A) Indicated strains were cultured in M63 minimal medium for ∼48 hours with aeration. Four or 10 U of DNaseI was added to wild type E264 cultures upon inoculation. B) Wild type and P_S12_-*bcpAIOB* strains were cultured in M63 minimal medium; photos were taken over the course of 48 hours.

Culturing wild type *B. thailandensis* in M63 minimal medium resulted in a dramatic autoaggregation phenotype in which bacteria aggregated and adhered to the walls of glass test tubes (Figure 5A). By contrast, the Δ*bcpAIOB* strain grew as a homogenous suspension of planktonic cells (Figure 5A), indicating that the *bcpAIOB* genes are required for autoaggregation. The Δ*bcpB* and Δ*bcpO* strains also grew planktonically (Figure 5A). Because TpsB family members are required for secretion of TpsA proteins to the bacterial surface and Western blot data confirmed that no BcpA protein could be detected in the Δ*bcpB* strain (Figure 3), the Δ*bcpB* strain was expected to have the same phenotype as the Δ*bcpAIOB* strain. The fact that the Δ*bcpO* strain failed to autoaggregate, however, indicates that the BcpO protein is required for BcpA function. Because the BcpA protein profile in the Δ*bcpO* mutant was identical to that of wild type bacteria, BcpO likely contributes to maturation events that occur during or after translocation across the outer membrane. Because CDI systems have so far been demonstrated to function only in interbacterial growth inhibition between CDI^+^ and CDI^−^ bacteria, these data represent the first demonstration of a phenotype for a strain defective for expression of genes encoding a (putative) CDI system in a homogeneous culture.

Efforts to complement the *bcpO* and *bcpB* genes were not successful in restoring the wild type autoaggregation phenotype. Specifically, expression of *bcpO* and *bcpB* from the P_S12_ promoter or the native promoter, P*_bcpA_*, at the *att*Tn7 site failed to restore autoaggregation in the Δ*bcpO* and Δ*bcpB* mutants, respectively (data not shown). To address the possibility that lack of autoaggregation in the Δ*bcpO* mutant was due to an unintended mutation other than the Δ*bcpO* mutation, we tested several independently constructed Δ*bcpO* mutant strains. All of these strains grew planktonically and failed to autoaggregate. Together, these data indicate that the *bcpAIOB* genes are required for autoaggregation, and they suggest that expression of the *bcpO* and *bcpB* genes from their native locus is critical for proper function of their gene products.

A strain in which expression of the *bcpAIOB* genes was controlled by the constitutively active P_S12_ promoter (P_S12_-*bcpAIOB*) also autoaggregated when cultured in M63 minimal medium, but with different kinetics and aggregation characteristics compared with wild type *B. thailandensis*. The P_S12_-*bcpAIOB* strain aggregated but was not adherent to the walls of the test tube at 24 hours, and by 48 hours, the adherent/aggregated bacteria had a smoother, more mucoid appearance (Figure 5B). These data indicate that controlled expression of the *bcpAIOB* genes (high in approximately 0.2% of the population and undetectable in the rest) is important for the wild type autoaggregation phenotype.

Autoaggregation and adherence to the walls of test tubes by *B. thailandensis* may be a form of biofilm. Because DNA has been shown to be an important component of many bacterial biofilms [20], we sought to determine if DNA contributed to the *B. thailandensis* autoaggregation phenotype. Addition of 4 U DNaseI decreased the amount of autoaggregation and addition of 10 U DNaseI completely abrogated autoaggregation (Figure 5A), causing the bacteria to grow planktonically. Extracellular DNA is therefore required for the autoaggregation phenotype. To quantify the amount of extracellular DNA in wild type cultures compared to Δ*bcpAIOB* cultures, supernatants were filter sterilized and analyzed by spectrophotometry. No difference in the quantity of DNA could be detected (data not shown), suggesting there may be variations in the quality of DNA present in the two cultures that mediate autoaggregation and/or that DNA–BcpA interactions are required for autoaggregation.

### *Burkholderia* BcpA-CT and BcpI proteins are toxin-immunity pairs that function in an allele-specific manner

**Figure 6:**
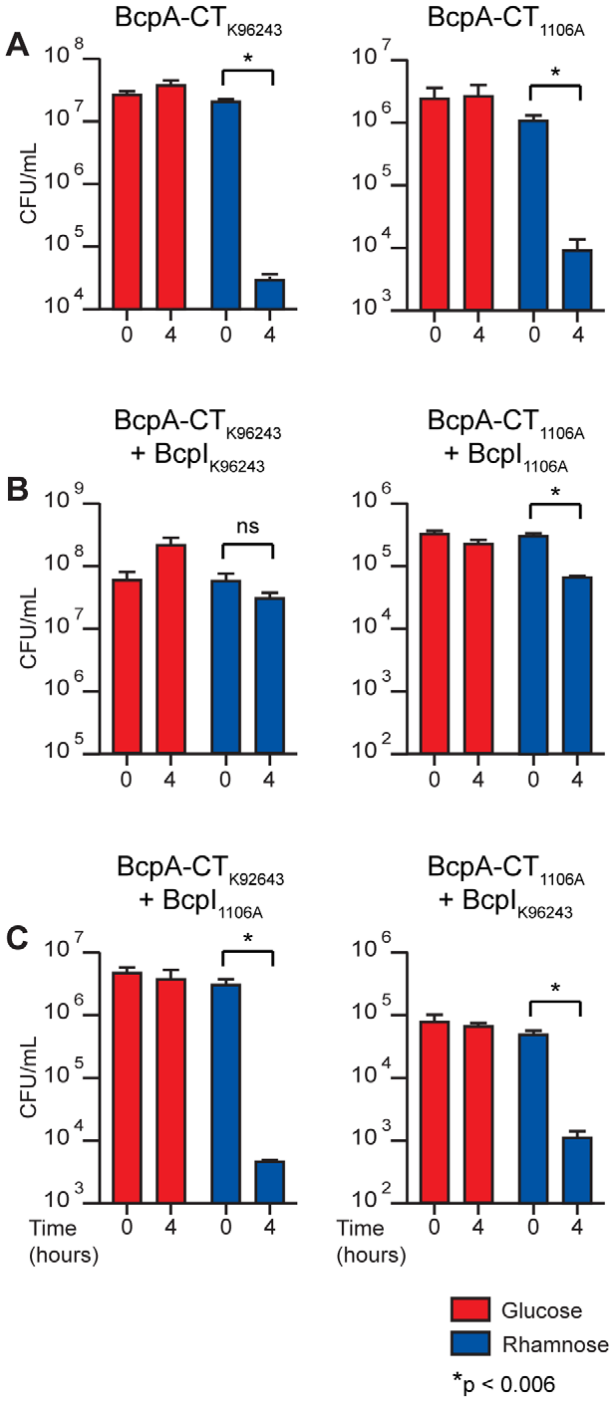
Intracellular toxicity of BcpA-CT proteins and protection by BcpI proteins. *B. thailandensis* producing BcpA-CT peptides with and without BcpI proteins from *B. pseudomallei*; specific combinations are indicated above each graph. Production of A) BcpA-CT_K96243_ (left panel) and BcpA-CT_1106A-2_ (right panel), B) BcpA-CT_K96243_ and BcpI_K96243_ (left panel) and BcpA-CT_1106A-2_ and BcpI_1106A-2_ (right panel), and C) BcpA-CT_K96243_ and BcpI_1106A-2_ (left panel) and BcpA-CT_1106A-2_ and BcpI_K96243_ (right panel) were repressed with 0.2% glucose (red bars) or induced with 0.2% rhamnose (blue bars). Error bars represent the mean ± 1 SEM.

Our inability to construct an *E. coli* strain producing the C-terminal ∼350 aa of BcpA from *B. pseudomallei* 1026b unless the cognate BcpI protein was also produced provided the first evidence that the C-terminal domains of CdiA/BcpA proteins are sufficient to cause toxicity when produced intracellularly [4]. Here, we constructed plasmids to encode the last ∼350 aa (including the Nx(E/Q)LYN motif) of BcpA from *B. pseudomallei* K96243 (BcpA-CT*_Bp_*_K96243_) and *B. pseudomallei* 1106A-2 (BcpA-CT*_Bp_*_1106A-2_) (with an added ATG at the 5′ end) 3′ to the rhamnose inducible promoter P*_rhaB_*. Overnight cultures of *B. thailandensis* containing these plasmids were supplemented with 0.2% glucose (to suppress P*_rhaB_*) and diluted into LSLB containing either 0.2% glucose or 0.2% rhamnose. Bacterial viability was monitored after four hours of culture at 37°C by counting colony forming units (cfu). The number of cfu/ml of *B. thailandensis* strains containing these plasmids cultured in medium containing 0.2% glucose was not altered after four hours (Figure 6A). By contrast, when cultured with 0.2% rhamnose to induce P*_rhaB_*, the number of cfu/ml of *B. thailandensis* strains containing the plasmids encoding BcpA-CT*_Bp_*_K96243_ and BcpA-CT*_Bp_*_1106A-2_ decreased by 3 and 2 logs, respectively (Figure 6A, left and right panels, respectively). These results indicate BcpA-CTs are sufficient to cause toxicity when produced intracellularly in *B. thailandensis*.

We next investigated the ability of BcpI to provide immunity to BcpA-CT-mediated intracellular toxicity. We constructed another set of plasmids encoding BcpI proteins from *B. pseudomallei* K96243 (BcpI*_Bp_*_K96243_) and *B. pseudomallei* 1106A-2 (BcpI*_Bp_*_1106A-2_) also under control of P*_rhaB_*. *B. thailandensis* containing these plasmids in combination with the BcpA-CT-encoding plasmids were cultured as described above. Again, bacterial viability was monitored after four hours. The number of cfu/ml of *B. thailandensis* harboring the plasmids encoding cognate BcpA-CT*_Bp_*_K96243_ and BcpI*_Bp_*_K96243_ increased 0.5 log when cultured with 0.2% glucose and decreased only slightly when cultured with 0.2% rhamnose (Figure 6B, left panel). Similarly, the number of cfu/ml of *B. thailandensis* harboring the plasmids encoding cognate BcpA-CT*_Bp_*_1106A-2_ and BcpI*_Bp_*_1106A-2_ decreased slightly when cultured with 0.2% glucose and decreased less than 1 log when cultured with 0.2% rhamnose (Figure 6B, right panel). While the decrease in cfu/ml of *B. thailandensis* containing the plasmids encoding BcpA-CT*_Bp_*_1106A-2_ and BcpI*_Bp_*_1106A-2_ observed when cultured with 0.2% rhamnose was statistically significant (p<0.006), the log fold change of this strain compared to the log fold change of *B. thailandensis* harboring the BcpA-CT*_Bp_*_1106A-2_ encoding plasmid alone was also statistically significant (p<0.0001) when cultured with 0.2% rhamnose. These data indicate cognate BcpI proteins are able to rescue the toxic phenotypes observed when BcpA-CTs are produced intracellularly. By contrast, the number of cfu/ml of *B. thailandensis* harboring plasmids encoding the non-cognate pair BcpA-CT*_Bp_*_K96243_ and BcpI*_Bp_*_1106A-2_ decreased by 3 logs when cultured with 0.2% rhamnose, but did not change when cultured with 0.2% glucose (Figure 6C, left panel); and *B. thailandensis* containing plasmids encoding the non-cognate pair BcpA-CT*_Bp_*_1106A-2_ and BcpI*_Bp_*_K96243_ decreased by 2 logs when cultured with 0.2% rhamnose, but did not change when cultured with 0.2% glucose (Figure 6C, right panel). These data indicate BcpI proteins mediate immunity to BcpA-CT intracellular toxicity in an allele-specific manner.

### Lack of evidence for BcpAIOB-mediated competition in liquid medium

**Figure 7:**
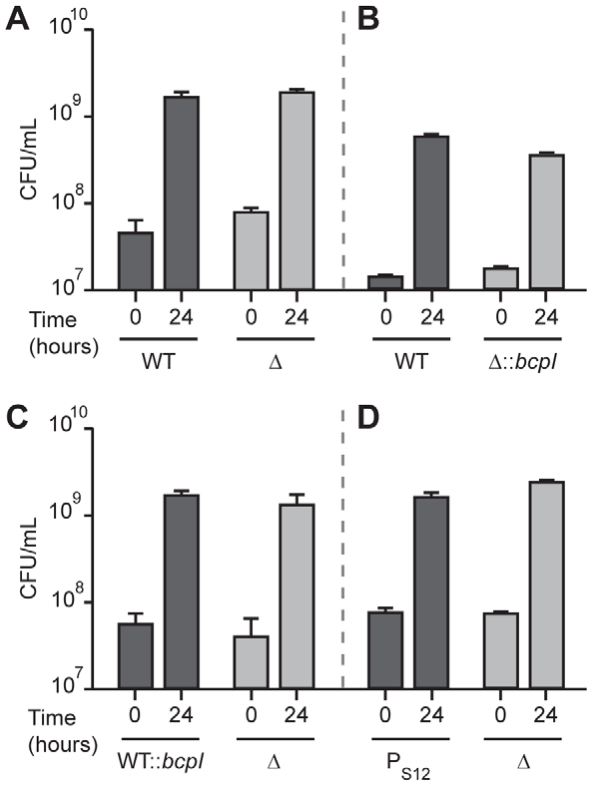
*bcpAIOB*-dependent competition does not occur in liquid medium. A) Wild type E264Cm^R^ (WT) co-cultured with Δ*bcpAIOB* (Δ). B) Wild type E264Cm^R^ (WT) co-cultured with Δ*bcpAIOB*::*bcpI*_E264_ (Δ::*bcpI*). C) Wild type E264::*bcpI*_E264_ (WT::*bcpI*) co-cultured with Δ*bcpAIOB* (Δ). D) Constitutive E264P_S12_-*bcpAIOB* (P_S12_) co-cultured with Δ*bcpAIOB* (Δ). For all assays, strains were cultured separately overnight, diluted to OD_600_ 0.2, mixed at a 1∶1 ratio, and co-cultured for 24 hours. Aliquots were taken at 0 hours and 24 hours, serially diluted, and plated with antibiotic selection to calculate the cfu/ml of each strain. Error bars represent the mean ± 1 SEM.

Previous work with *E. coli* demonstrated that *E. coli*-type CDI systems function in interbacterial competition in liquid medium when the *cdiBAI* genes are expressed from a constitutive or inducible promoter [1], [4]. Additionally, it was demonstrated that expression of the cognate immunity gene in target bacteria was protective against CDI [4]. To determine if the *bcpAIOB* genes also mediate interbacterial competition and if *bcpI* provides protection, similar competition assays were performed. Wild type inhibitor (E264Cm^R^) and Δ*bcpAIOB* mutant target bacteria were mixed at a 1∶1 ratio and cultured in LSLB broth at 37°C for 24 hours without antibiotic selection. Wild type bacteria were also cultured at a 1∶1 ratio with Δ*bcpAIOB* bacteria constitutively expressing the cognate immunity gene (E264Δ*bcpAIOB*::*bcpI*_E264_) under the same conditions. After 24 hours, the cultures were serially diluted and plated on selective media to determine the number of cfu of each strain. In the competition between wild type and Δ*bcpAIOB* mutant bacteria, both strains grew equally (Figure 7A), and therefore no competitive advantage was observed for the wild type strain in this assay. Both strains also grew equally in the competition between wild type and Δ*bcpAIOB*::*bcpI*_E264_ bacteria (Figure 7B). Since our expression data indicated that *B. thailandensis bcpAIOB* genes are expressed in only about one in one thousand wild type bacterial cells cultured in liquid medium (Figure 4), we hypothesized two non-mutually exclusive reasons for the lack of apparent competition between wild type bacteria and the Δ*bcpAIOB* mutant. 1) In addition to inhibiting Δ*bcpAIOB* mutant bacteria, the wild type bacteria expressing their *bcpAIOB* genes might also inhibit the growth of wild type bacteria not expressing their *bcpAIOB* genes because these bacteria would not be producing BcpI, and hence both wild type and Δ*bcpAIOB* bacteria would be inhibited to the same extent. 2) Regardless of the susceptibility of wild type bacteria to growth inhibition by other wild type bacteria, the number of Δ*bcpAIOB* mutant bacteria inhibited by wild type bacteria may be insignificant because of the low number of wild type bacteria expressing their *bcpAIOB* genes.

To test the first hypothesis, we constructed a strain constitutively expressing the cognate *bcpI* gene (from *B. thailandensis* E264) in a wild type E264 background (E264::*bcpI*_E264_). Constitutive expression of *bcpI*_E264_ in the wild type strain did not alter the results of the competition assay; both strains again grew equally (Figure 7C), suggesting that wild type bacteria expressing *bcpAIOB* were not inhibiting wild type bacteria that were not expressing *bcpAIOB* to an appreciable level. To test the second hypothesis, we performed a competition experiment with the strain expressing *bcpAIOB* from the P_S12_ promoter (P_S12_-*bcpAIOB*) and Δ*bcpAIOB* bacteria. Again, both strains grew equally (Figure 7D), suggesting expression of *bcpAIOB* in every wild type bacterium does not lead to growth inhibition of mutant target bacteria in liquid medium. Collectively, these data indicate that expression of *bcpAIOB* from either the native promoter or a constitutively active promoter (in single copy on the chromosome) is not sufficient to cause interbacterial competition against Δ*bcpAIOB* target bacteria when mixed at a 1∶1 ratio in liquid medium.

### The *bcpAIOB*-encoded CDI system mediates interbacterial competition on solid medium

#### Initial characterization of BcpAIOB-mediated CDI on solid medium

**Figure 8:**
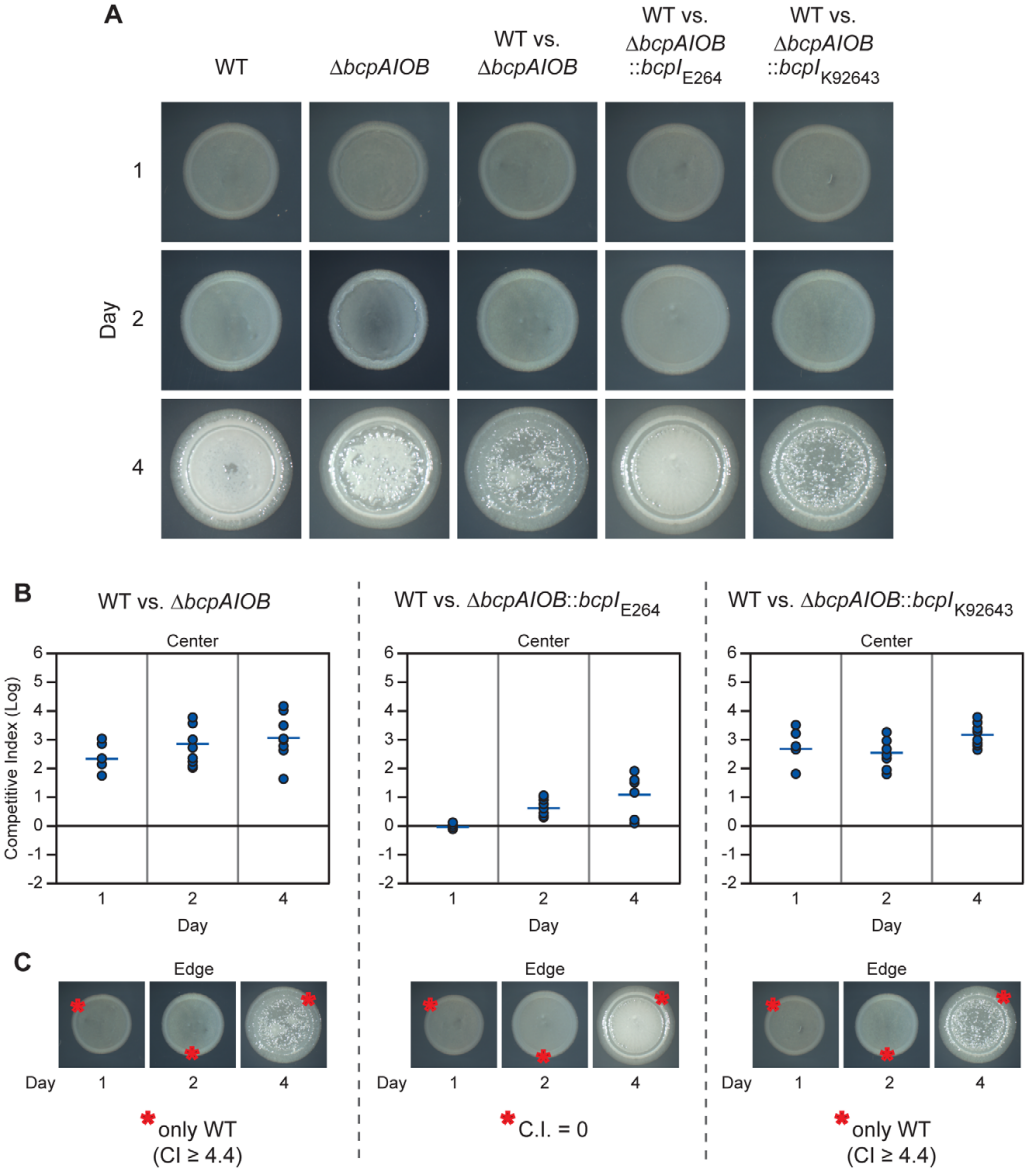
*bcpAIOB*-mediated contact dependent growth inhibition on solid medium. A) Colony biofilms of indicated strains and competitions were observed for four days by microscopy. B) Samples from the center of each colony biofilm competition were taken on each day and plated with antibiotic selection to determine the competitive index (C.I.) of wild type E264Cm^R^ (WT) bacteria compared to Δ*bcpAIOB* (left panel), Δ*bcpAIOB*::*bcpI*_E264_ (middle panel), and Δ*bcpAIOB*::*bcpI*_K96243_ (right panel). C) Samples from a single location along the leading edge of each colony biofilm (as indicated by the red asterisks(*)) were plated as in B. In cases where only wild type bacteria were recovered, the actual C.I. is greater than or equal to the value stated.

We set out to determine if the *bcpAIOB* gene products affected growth and interbacterial competition on solid medium. To begin, overnight cultures of wild type and Δ*bcpAIOB* strains were diluted to OD_600_ = 0.2 and 20 µl of culture was spotted onto LSLB agar to initiate colony biofilm formation and observed for four days. Competition colony biofilms (strains mixed at a 1∶1 ratio) were plated at the same dilution and also observed for four days (Figure 8A). The initial boundaries of the colony biofilms were apparent by day 1. Between two and four days, the bacteria began to migrate outward, giving rise to a leading edge that was distinct from the initial boundary, thereby increasing the diameter of the colony biofilm. The wild type and Δ*bcpAIOB* mutant colony biofilms exhibited distinct morphological properties after four days. However, both strains grew past the initial boundary and migrated equal distances after four days, from ∼9 mm to 11 mm, indicating that *B. thailandensis* is capable of moving across a solid surface and that the *bcpAIOB* genes are not required for this phenotype. Each colony biofilm formed from a heterogeneous population (the competition colony biofilms) also had a distinct morphology, but again the bacteria were able to migrate past the initial boundary.

Bacteria were picked from the center of the competition colony biofilms on days 1, 2, and 4 post inoculation using a sterile pipette tip, suspended in PBS, diluted, and aliquots were plated on LSLB agar containing Cm to select for wild type bacteria and LSLB agar containing Km to select for Δ*bcpAIOB* mutant bacteria, in order to determine the competitive index of CDI^+^ inhibitors compared to CDI^−^ targets. Wild type (E264Cm^R^) bacteria outcompeted the Δ*bcpAIOB* mutant by approximately 2.5 logs at one day and this increased to ∼3 logs at four days (Figure 8B, left panel). Constitutive expression of the cognate *bcpI* gene in the Δ*bcpAIOB* mutant strain (E264Δ*bcpAIOB*::*bcpI*_E264_) provided protection, as this strain competed equally with the wild type strain at one day and was only slightly outcompeted at two and four days (Figure 8B, middle panel). Constitutive expression of a heterologous *bcpI* gene (from *B. pseudomallei* K92643) however, did not provide protection to the Δ*bcpAIOB* mutant strain (E264Δ*bcpAIOB*::*bcpI*_K92643_), which was outcompeted by approximately 2.5 logs at one day and ∼3 logs at four days (Figure 8B, right panel). Together, these data show that the *bcpAIOB* chromosomally-encoded CDI system in *B. thailandensis* mediates interbacterial competition on a solid agar surface and that immunity to interbacterial CDI is allele-specific.

On each day (1, 2, and 4 post inoculation), samples were also taken from a single location along the leading edge of each colony biofilm to determine the competitive index of CDI^+^ inhibitors compared to CDI^−^ targets. Because the colony biofilms increased in diameter by 0.5–1.0 mm each day, the sampled regions on days 2 and 4 contained bacteria that had migrated outward from the initial boundary on day 1. Remarkably, no Δ*bcpAIOB* bacteria were recovered from the leading edge of competitions between wild type and Δ*bcpAIOB* bacteria (Figure 8C, left panel) or wild type and Δ*bcpAIOB*::*bcpI*_K92643_ bacteria (Figure 8C, right panel) at any time point (the competitive index is therefore greater than or equal to 4.4), suggesting CDI-mediated competition is very strong at the leading edge. Constitutive expression of the cognate *bcpI* gene in Δ*bcpAIOB* mutant bacteria (E264Δ*bcpAIOB*::*bcpI*_E264_) abrogated competition, as this strain was present in equal numbers to wild type bacteria on each day tested (Figure 8C, middle panel). Collectively, these data indicate that the majority of BcpAIOB-mediated competition occurs prior to day 1, and suggest competition is more robust at the leading edge than in the center of the colony biofilm.

#### Analysis of early time points of BcpAIOB-mediated CDI

**Figure 9:**
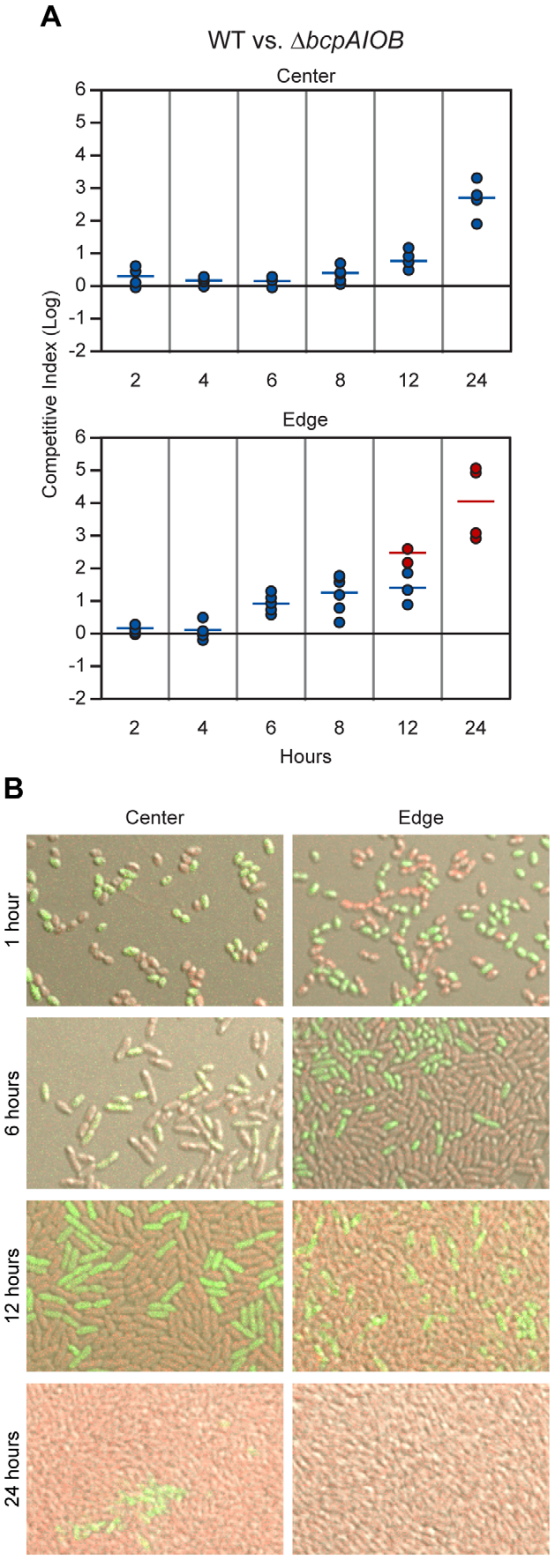
Analysis of 24–hour time course of CDI-mediated competition. A) Wild type E264Cm^R^ (WT) bacteria and Δ*bcpAIOB* mutant bacteria were co-incubated on solid medium. Samples were taken from the center (top panel) and edge (bottom panel) at the indicated times. The C.I. was determined as in Figure 8B. Red data points indicate only wild type bacteria were recovered, and the actual C.I. is therefore greater than or equal to the represented value. B) Microscopy of E264P_S12_-*rfp* and E264Δ*bcpAIOB*P_S12_-*gfp* mixed at a 1∶1 ratio in the center (left column) and edge (right column) of colony biofilms at the indicated times.

To gain a better understanding of BcpAIOB-mediated CDI, we conducted a 24–hour time course competition between wild type and Δ*bcpAIOB* bacteria. Competition was apparent at six hours at the edge of the colony biofilm, and by 12 hours, no Δ*bcpAIOB* bacteria were recovered in about half of the samples (the competitive index is therefore greater than or equal to the represented value, red data points) (Figure 9A, bottom panel). By contrast, in the center of the colony biofilm, competition was not apparent until 12 hours, and Δ*bcpAIOB* bacteria were still recovered at 24 hours, albeit as a very low proportion of the population (Figure 9A, top panel). These results indicate that the effects of CDI can be observed earlier at the edge of the colony biofilm than in the center.

To investigate the difference in competition at the center and edge of the colony biofilm in more detail, we visualized the colony biofilms by live-image microscopy. One hour after plating the bacteria, the initial edge contained densely packed bacteria, whereas the center of the colony biofilm contained sporadically distributed bacteria, most of which were not in contact with any other bacteria (Figure S4). By 12 hours, bacteria in the center of the colony biofilm had divided to the point that most were in contact with other bacteria (Figure S4). The difference in density of bacteria over time within the colony biofilm therefore, may explain the difference in competition observed between the center and the edge.

To distinguish wild type and mutant bacteria within the colony biofilms, we labeled wild type bacteria with RFP (E264::P_S12_-*rfp*) and Δ*bcpAIOB* bacteria with GFP (E264Δ*bcpAIOB*::P_S12_-*gfp*) and visualized the colony biofilms by confocal microscopy. (Note that after addition of a cover slip to the top of the colony biofilm, the original architecture was disrupted, and bacteria were moderately displaced (i.e. spread out) from their original location. For authentic architecture of the colony biofilms, refer to Figure S4.) After one hour of co-incubation on agar, the bacteria were cocci-shaped (Figure 9B, first row). (Again, note that bacteria from the edge of the colony biofilms particularly at one hour were spread out from their original location when visualized for this assay. Prior to manipulation, these bacteria were in close association with one another.) Strikingly, after six hours on a solid surface, the morphology of the bacteria changed from cocci to more rod-shaped (Figure 9B, second row), and by 12 hours, the bacteria were long rods (Figure 9B, third row). These data suggest the bacteria undergo a change in gene expression and cell morphology in response to either their change in environment upon plating on solid medium or due to the change from stationary phase growth (in liquid medium prior to plating) to log phase on solid medium. Interestingly, some Δ*bcpAIOB*::P_S12_-*gfp* bacteria at the edge at six hours still appear to be cocci-shaped, suggesting they were previously growth inhibited by wild type bacteria and therefore unable to transition to rod-shaped, whereas this was not observed in colony biofilms containing only wild type bacteria (Figure S5).

With respect to the proportion of wild type (RFP^+^) and Δ*bcpAIOB* (GFP^+^) bacteria, both were present in equal numbers in the center and edge of the colony biofilm at one hour (Figure 9B, first row). At six hours, RFP^+^ and GFP^+^ bacteria were again detected in equal numbers in the center of the colony biofilm, while a greater proportion of wild type (RFP^+^) bacteria than Δ*bcpAIOB* (GFP^+^) bacteria were present along the edge (Figure 9B, second row). By 12 hours, the proportion of RFP^+^ bacteria had increased in both the center and edge of the colony biofilm (Figure 9B, third row), and after 24 hours of co-incubation, only RFP^+^ bacteria were detected along the edge, while some GFP^+^ bacteria could still be detected in some samples from the center of the colony biofilm, a representative of which is shown in Figure 9B. As a control, we mixed wild type strains expressing *rfp* or *gfp* (E264::P_S12_-*rfp* and E264::P_S12_-*gfp*) at a 1∶1 ratio, plated them on agar, and looked microscopically at 1, 6, 12, and 24 hours. For all time points, RFP^+^ and GFP^+^ bacteria were present in equal numbers (Figure S5). These data support the competition data presented in Figure 9A indicating that competition can be observed at earlier time points and to a greater extent at the edge of the colony biofilm compared to the center.

Together, these data show that wild type *B. thailandensis* is able to outcompete otherwise isogenic Δ*bcpAIOB* mutant bacteria via CDI when mixed on an agar surface in the center of, and to an even greater extent, at the initial boundary of a colony biofilm within 24 hours. Immunity to interbacterial CDI is allele-specific, as only expression of the cognate immunity gene and not a heterologous immunity gene provided protection to target bacteria. Bacteria in the center of the colony biofilm were not subject to CDI initially because they were not in direct contact with other bacteria. Upon sufficient growth to allow direct interbacterial interactions, some mutant bacteria however, were still surrounded only by Δ*bcpAIOB* bacteria, and therefore competition was not “complete” in the center of the colony biofilm. By contrast, at the initial edge of the colony biofilm, wild type and mutant bacteria were in direct contact immediately upon plating, and nearly every mutant bacterium was in contact with a wild type bacterium. Competition in this context took four to six hours to become apparent, suggesting a change in gene expression must occur to initiate CDI, and was “complete” by 12–24 hours.

#### Investigation of expression of the *bcpAIOB* genes on solid medium

When cultured in liquid medium, *bcpAIOB* gene expression was detected in only approximately one in one thousand bacteria (Figure 4). We hypothesized that expression occurred in a much greater proportion of the population on solid medium, given the level of competition that was observed in the colony biofilms (Figures 8 & 9). To investigate the expression of the *bcpAIOB* genes in bacteria in a colony biofilm, we performed microscopy and flow cytometry using our P*_bcpA_*-*gfp* strain as a reporter. Bacteria were scraped from the center and edge of colony biofilms on solid agar at 2, 4, 6, 8, 12, 24, and 48 hours post-plating, suspended in PBS, and analyzed. At no time point were any GFP^+^ bacteria detected by either microscopy or flow cytometry (data not shown). However, our data indicate that BcpAIOB-mediated CDI had occurred under these conditions (Figures 8 & 9) and therefore the *bcpAIOB* genes must have been expressed. These data suggest therefore, that expression from the P*_bcpA_* promoter in bacteria grown on a solid surface is either too weak or too transient to be detected by our assays.

**Figure 10:**
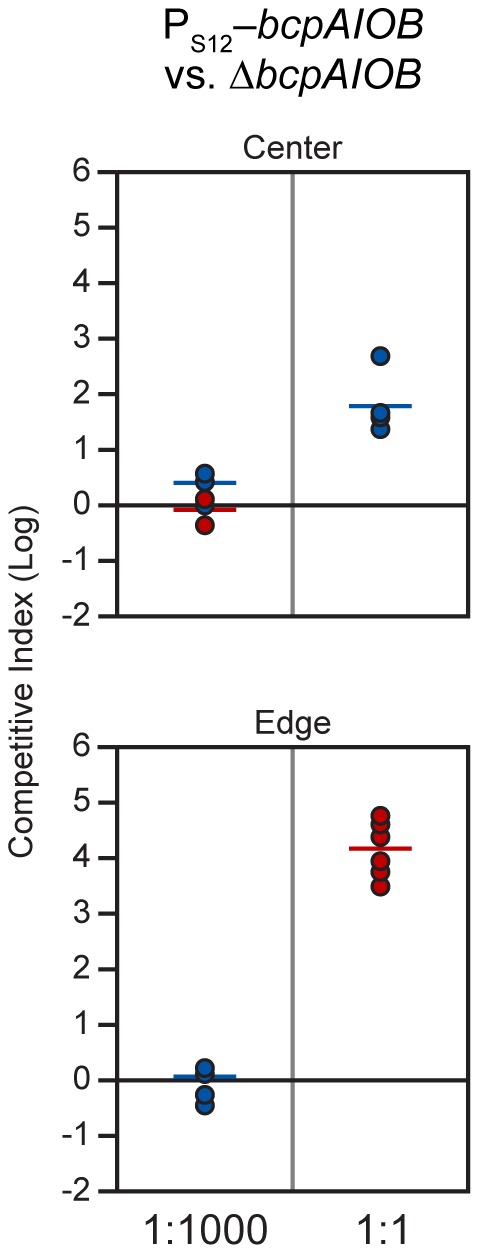
P_S12_-*bcpAIOB*-mediated contact dependent growth inhibition. E264P_S12_-*bcpAIOB* (P_S12_) co-cultured with Δ*bcpAIOB* (Δ) mutant bacteria at a 1∶1,000 ratio (left column) or a 1∶1 ratio (right column) on solid medium. The C.I. was determined for the center (top panel) and edge (bottom panel) of the colony biofilm as described in Figure 8B. Red data points indicate only wild type bacteria were recovered, and the actual C.I. is therefore greater than or equal to the represented value.

Because we could not detect *bcpAIOB* expression in cells during growth in colony biofilms, we developed an alternative method to determine the proportion of BcpAIOB^+^ cells required to outcompete BcpAIOB^−^ cells on a solid surface. We performed competition experiments with our strain in which the *bcpAIOB* genes were expressed constitutively (P_S12_-*bcpAIOB*) mixed at a 1∶1,000 ratio with Δ*bcpAIOB* bacteria to simulate expression similar to that in liquid medium. The competitive index did not change in either the center or the edge of the colony biofilm after 24 hours (Figure 10), indicating no competition occurred. When we mixed P_S12_-*bcpAIOB* and Δ*bcpAIOB* bacteria at a 1∶1 ratio to simulate 100% of the “wild type” population activating the *bcpAIOB* genes, P_S12_-*bcpAIOB* bacteria outcompeted Δ*bcpAIOB* mutant bacteria by nearly 2 logs in the center of the colony biofilm and completely outcompeted the mutant along the leading edge after 24 hours (Figure 10), similar to competition between wild type and Δ*bcpAIOB* bacteria (Table 2). Collectively, these results strongly suggest that expression of the *bcpAIOB* genes occurs in a greater proportion of cells than one in one thousand, if not 100% of the population, on solid medium – indicating a change in gene expression in response to the solid surface colony biofilm environment.

**Table 2:** CDI activity of *B. thailandensis* strains.

Inhibitor strain^1^	Center^2^	Edge^2^
E264	2.36	≥4.38*
E264P_S12_-*bcpAIOB*	1.75	≥4.19*
E264Δ*bcpO*	1.23	1.66
E264Δ*bcpO*::*bcpO*	1.51	≥4.53*

1 Mixed 1∶1 with E264Δ*bcpAIOB* on agar.

2 Average C.I. (log) at 24 hours.

* Only inhibitor strain recovered.

### *bcpO* is required for complete CDI-mediated interbacterial competition

**Figure 11:**
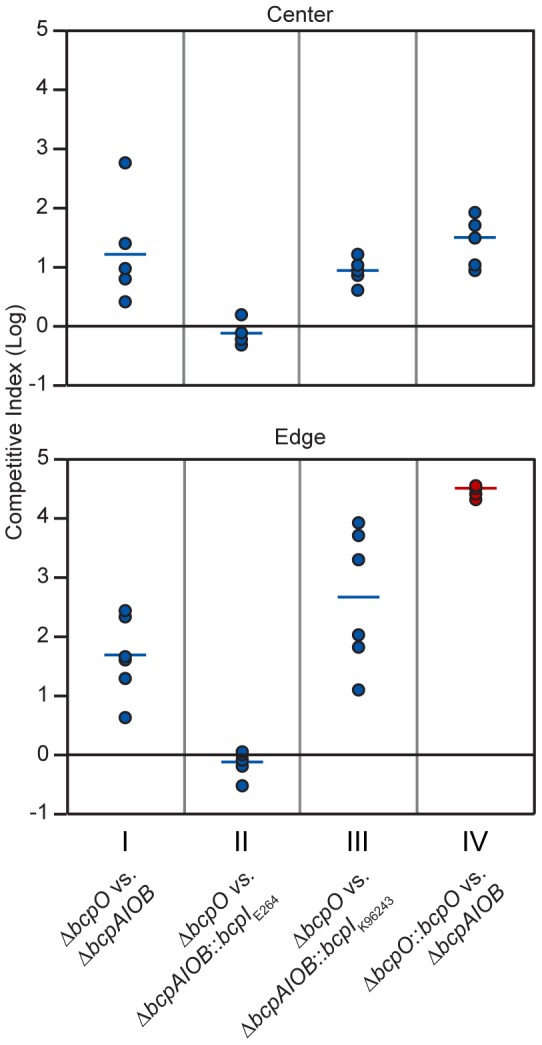
Δ*bcpO*-mediated contact dependent growth inhibition. Cultures of bacteria were mixed at a 1∶1 ratio and colony biofilms were plated on solid LSLB agar. Samples were taken from the center (top panels) and leading edge (bottom panels) of the colony biofilms at day 1 and plated with antibiotic selection to determine the C.I. of Δ*bcpO* compared to Δ*bcpAIOB* (column I), Δ*bcpO*::*bcpO*_E264_ compared to Δ*bcpAIOB* (column II), Δ*bcpO* compared to Δ*bcpAIOB*::*bcpI*_E264_ (column III), and Δ*bcpO* compared to Δ*bcpAIOB*::*bcpI*_K96243_ (column IV). Red data points indicate only wild type bacteria were recovered, and the actual C.I. is therefore greater than or equal to the represented value.

To determine the contribution of *bcpO* to BcpAIOB-mediated CDI, we conducted a competition between Δ*bcpO* bacteria and Δ*bcpAIOB* bacteria. In the center of the colony biofilm, Δ*bcpO* bacteria outcompeted Δ*bcpAIOB* bacteria by ∼1 log at 24 hours (Figure 11, top panel, column I) and along the leading edge of the colony biofilm, Δ*bcpO* bacteria outcompeted Δ*bcpAIOB* bacteria by ∼1.5 logs at 24 hours (Figure 11, bottom panel, column I). For both locations, competition by the Δ*bcpO* strain was severely reduced compared to competition by wild type bacteria (Table 2). To determine if the small competitive advantage displayed by the Δ*bcpO* strain was in fact due to CDI, we competed Δ*bcpO* bacteria with Δ*bcpAIOB* mutant bacteria constitutively expressing the cognate *bcpI* gene (E264Δ*bcpAIOB*::*bcpI*_E264_) or a heterologous *bcpI* gene (E264Δ*bcpAIOB*::*bcpI*_K96243_). The competitive index for competition between the Δ*bcpO* strain and E264Δ*bcpAIOB*::*bcpI*_E264_ was zero in the center and along the leading edge of the colony biofilm at 24 hours (Figure 11, column II), indicating constitutive expression of *bcpI*_E264_ in Δ*bcpAIOB* mutant bacteria is protective against CDI by the Δ*bcpO* strain. However, constitutive expression of *bcpI*_K96243_ in Δ*bcpAIOB* mutant bacteria was not protective, as Δ*bcpO* bacteria outcompeted E264Δ*bcpAIOB*::*bcpI*_K96243_ bacteria by ∼1 log in the center and ∼2 logs along the leading edge of the colony biofilm at 24 hours (Figure 11, column III). Complemention of the Δ*bcpO* strain with a copy of *bcpO* expressed constitutively (E264Δ*bcpO*::*bcpO*) only slightly increased CDI activity in the center of the colony biofilm but fully restored activity at the edge, i.e. Δ*bcpAIOB* bacteria were completely outcompeted in this location (Figure 11, column IV, Table 2). Lack of restoration of autoaggregation and partial restoration of CDI by the *bcpO* complementation strain underscores the complexity of the *bcpAIOB* system and the role of BcpO in interbacterial CDI.

Together, our data indicate that the Δ*bcpO* strain has a greater than ten–fold defect in CDI-mediated interbacterial competition in the center of the colony biofilm and at least a thousand–fold defect along the leading edge compared to wild type bacteria after 24 hours (Table 2). BcpO, therefore, plays a substantial and critical role in CDI-mediated interbacterial competition in *B. thailandensis*.

## Discussion

CDI has so far been demonstrated only in *E. coli*, the species in which it was discovered, and *Dickeya dadantii*, a phytopathogenic bacterium that infects a variety of crop plants [1], [4]. Although genetic loci in six *Burkholderia* strains were predicted to encode CDI systems based on the presence of small (∼300 bp) ORFs immediately 3′ to genes predicted to encode TpsA proteins, the gene order in these loci was different than that of the *E. coli cdiAIB* genes (and all other putative CDI system-encoding loci) and the motif separating the conserved and variable regions of the predicted TpsA protein was NxxLYN rather than VENN [4]. We showed in this study that the *Burkholderia bcpAIOB* genes do in fact encode proteins that function in many ways like the CDI system of *E. coli*; the BcpA-CTs are toxic when produced intracellularly, the *bcpI* genes confer immunity in an allele-specific manner, and wild type *B. thailandensis* can outcompete a Δ*bcpAIOB* mutant if the mutant does not express the cognate immunity gene. *B. thailandensis* is therefore the third species in which CDI has been demonstrated. Moreover, together with the previous observations, our results indicate that *Burkholderia bcpAIOB* genes define novel classes of both CDI and TPS systems. We also showed in this study that the *Burkholderia bcpAIOB* genes are required for autoaggregation and adherence to an abiotic surface, and that they are expressed in a probabilistic manner when the bacteria are cultured in liquid medium, two phenotypes not previously ascribed to CDI or CDI system-encoding genes.

The TPS pathway is one of the simplest mechanisms for the secretion of proteins to the surface of Gram-negative bacteria. The paradigm, based primarily on studies of the FHA/FhaC proteins of *Bordetella* species and the HMW1/HMW1B proteins of *Haemophilus influenzae*, states that the large β-helical exoprotein (the TpsA family member) is translocated across the cytoplasmic membrane by the general Sec pathway and then requires only one protein, the TpsB family member, for translocation across the outer membrane [2], [3]. Experimental support for the sufficiency of the TpsB protein in outer membrane translocation of the TpsA protein was recently obtained in a study using purified FhaC, liposomes, and a polypeptide corresponding to the N-terminal 370 aa of FHA [21]. Our bioinformatic analysis identified a small ORF located 5′ to *bcpB* in most *Burkholderia*-type putative CDI protein-encoding loci, which we named *bcpO*. BcpO of *B. thailandensis* E264 is predicted to be a lipoprotein that localizes to the inner leaflet of the outer membrane and deletion of *bcpO* resulted in loss of autoaggregation (identical to deletion of the entire *bcpAIOB* operon) and significantly reduced CDI activity. BcpA appeared to be produced and exported across the outer membrane in the *bcpO* mutant, based on the fact that BcpA was not degraded, as assessed by immunoblot, and was capable of mediating a very low level of CDI. Unfortunately, our attempts to visualize BcpA on the surface of wild type and Δ*bcpO* mutant bacteria were unsuccessful (data not shown). Based on the phenotypes of the *bcpO* mutant and the predicted cellular location of BcpO, we hypothesize that BcpO is involved in efficient, export across the outer membrane, maturation of BcpA into a functional protein, release of BcpA from the cell surface, and/or sensing interbacterial interactions. Regardless of its role, our data indicate that the *Burkholderia* BcpAIOB proteins define a novel class of TPS system that requires an additional small protein, BcpO, to produce a fully functional TpsA protein.

TpsB proteins are members of the Omp85-TpsB superfamily, which includes BamA (the main component of the Bam complex that inserts β-barrel proteins into the outer membranes of Gram-negative bacteria), Tob55/Sam50 (which inserts proteins into the outer membranes of mitochondria), and Toc75 (which inserts proteins into the outer membranes of chloroplasts) [22]. In addition to BamA, the Bam complex contains four lipoproteins, BamB, C, D, and E, that localize to the inner leaflet of the outer membrane and play important but mostly unknown roles in outer membrane protein assembly [22]. The BcpAIOB system of *B. thailandensis* E264 may function in an analogous manner to the Bam complex, as it appears to require at least one predicted periplasmic lipoprotein for proper secretion or maturation of its substrate. Curiously, some classes of predicted CDI systems in *Burkholderia* spp contain BcpO proteins that do not have signal sequences and that vary in an allele-specific manner with their cognate BcpA and BcpI proteins. Whether these proteins function similarly to BcpO of *B. thailandensis* E264 or perform completely different functions is unknown. These systems may represent yet additional variation of the TPS and CDI paradigms.

Our P*_bcpA_*-*gfp* studies showed that when *B. thailandensis* is cultured in liquid medium (either LSLB or M63), expression of *bcpAIOB* is high in approximately 0.2% of the bacteria and undetectable in the rest. The only other strain for which expression of CDI system-encoding genes has been demonstrated is *E. coli* EC93, which, in contrast to other strains that have been investigated, appears to express the *cdiBAI* genes constitutively [4]. Our result suggests that, in liquid medium under the conditions tested, *bcpAIOB* gene expression is controlled in a probabilistic manner. Several cellular differentiation processes in organisms ranging from bacteria to humans are controlled probabilistically and transiently [23], [24], [25], one of the best understood being the development of competence in *Bacillus subtilis*. When starved for nutrients, a minority of *B. subtilis* cells in a population express genes required for DNA uptake (competence), while the rest commit to sporulation [26]. Regulation of competence genes in *B. subtilis* involves an excitable core module containing both positive and negative feedback loops [27]. We expect that equally complex regulatory circuits control *bcpAIOB* expression in *Burkholderia*, and our future experiments will be aimed at identifying and characterizing the systems involved.

When cultured in M63 minimal medium, wild type *B. thailandensis* aggregated and adhered to the walls of glass test tubes. This phenotype required BcpAIOB as the Δ*bcpAIOB*, Δ*bcpO*, and Δ*bcpB* mutants grew planktonically. This is the first demonstration of a phenotype for any CDI^−^ strain other than susceptibility to growth inhibition by CDI^+^ counterpart bacteria. Although we did not formally test for biofilm formation, Schwarz *et al.* showed that *B. thailandensis* forms a biofilm in a flow chamber model [28]. Our data suggest that the BcpAIOB proteins contribute to biofilm formation and therefore their true role in nature may not be (just) mediating interbacterial competition. Interestingly, the conditions that promote *B. thailandensis* autoaggregation are the same conditions in which expression of the *bcpAIOB* genes occurs in only about 0.2% of the bacterial cells. Treating cultures with DNaseI abrogated autoaggregation, suggesting that DNA may be an essential component of an extracellular matrix that holds aggregated cells together. This observation suggested the intriguing hypothesis that CDI contributes to biofilm formation by killing a small proportion of cells in the population such that their released DNA can be used for extracellular matrix formation. However, the strain expressing *bcpAIOB* from the strong, constitutive P_S12_ promoter also aggregated, albeit with different kinetics. In this population, all cells should produce BcpI and therefore should be immune to CDI, although we cannot rule out the possibility that strong, constitutive expression of the *bcpAIOB* locus alters the functionality of the individual gene products. Moreover, we detected extracellular DNA in cultures of both wild type and Δ*bcpAIOB* bacteria, indicating that extracellular DNA is required but not sufficient for autoaggregation. These observations underscore the complexity of the aggregation/biofilm phenotype and indicate that determining the role of BcpAIOB in this microbial lifestyle will require an extensive amount of additional investigation.

By contrast to what was observed for *B. thailandensis* cultured in liquid, BcpAIOB-dependent interbacterial competition was easily observed when the bacteria were grown on a solid surface. Within the colony biofilm, wild type bacteria outcompeted Δ*bcpAIOB* mutants by ∼2.5 logs in the center and completely (≥4.4 logs) at the edge after 24 hours of co-incubation. Examination of earlier time points indicated that competition occurred as early as 12 hours in the center and six hours at the edges. Live-cell imaging provided an explanation for the difference in competition at the two sites; bacteria in the center of the colony biofilm were just beginning to contact each other between 6 and 12 hours, while bacteria at the edges were in contact immediately upon plating. The efficiency of the competition when bacteria were in contact however, suggested that the *bcpAIOB* genes must be expressed in more than ∼0.2% of wild type bacteria under these conditions. The fact that bacteria transitioned from coccobacilli to long rods after being moved from stationary phase growth in broth to the solid surface provided evidence for a change in gene expression, but we were unsuccessful in our attempts to detect GFP^+^ bacteria when the P*_bcpA_-gfp* strain was used to form colony biofilms. However, the strain expressing *bcpAIOB* from the P_S12_ promoter was able to outcompete the Δ*bcpAIOB* strain when the colony biofilms were initiated with the bacteria present at a 1∶1 ratio but not when present at a 1∶1,000 ratio. Together, these data suggest that expression of the *bcpAIOB* operon is induced in a majority of the bacteria within a few hours after plating on agar. The competitive index did not increase between 24 and 96 hours in the center of the colony biofilm where there were still Δ*bcpAIOB* mutants present, however, suggesting that the early activation of *bcpAIOB* gene expression was only transient. Moreover, the fact that streaking the strain containing a P*_bcpA_*-*lacZ* fusion resulted in a heterogeneous population of dark and light blue colonies indicates that the signal that induces *bcpAIOB* gene expression is not solely the solid surface environment. We are currently investigating the possibility that *bcpAIOB* gene expression is induced in response to both environmental cues and recognition of neighboring bacteria, including sensing whether those neighboring bacteria have BcpA proteins on their surface.

By contrast with our observations with *B. thailandensis*, CDI in *E. coli* occurs in liquid medium [1]. In strain EC93, the rat fecal isolate in which CDI was discovered, the *cdiBAI* genes are expressed constitutively [1]. In human uropathogenic *E. coli* strain 536, *cdiBAI* expression was not detected under standard laboratory growth conditions [4]. To measure CDI activity in 536, or in laboratory K12 strains of *E. coli*, therefore, the *cdiBAI* genes were expressed from an inducible promoter on a high copy number plasmid [1], [4]. Moreover, it was shown that production of capsule or P or S pili in the target bacteria blocked CDI [1]. These caveats raised questions about the biological relevance of CDI as observed under these conditions. In light of our findings, the possibilities that *cdiBAI* genes in *E. coli* and other bacteria are expressed in a probabilistic and transient manner and that they contribute to aggregation and/or biofilm formation are compelling hypotheses to test.

Another apparent difference between *Burkholderia*-type CDI systems and *E. coli*-type CDI systems is the presence of one or more “orphan *cdiA-CT/cdiI* modules” located 3′ to *cdiBAI* genes [5]. Comparative genome analyses suggest that these modules may move within or between bacteria into the functional *cdiBAI* locus, changing the allele that is expressed, and hence they may contribute to the diversity of *cdiBAI* alleles within a population and even within a clonal population [5]. We searched specifically for *bcpA-CT/bcpI* modules in bacteria containing *Burkholderia*-type CDI systems but found no evidence for their existence. Diversity of CDI systems amongst *Burkholderia* must, therefore, occur by a different mechanism.

Our experiments, aimed at providing an initial characterization of the function of the *bcpAIOB* gene products and determining their contribution to aggregation and interbacterial competition, used wild type and mutant derivatives of *B. thailandensis* strain E264. As environmental saprotrophs, however, *Burkholderia* species share their habitats with a plethora of other bacteria, as well as viruses and eukaryotes. How CDI is used in this environment and whether it occurs only in an intra-species manner or also in an inter-species manner is not known. Our bioinformatic analysis identified 58 *bcpAI(O)B* loci and 41 different alleles. Some strains have multiple alleles and several alleles are present in multiple strains. Notably, one allele that is present in two different *B. pseudomallei* strains (1106A-1 and BCC215-2) is also present in *B. gladioli* (strain BSR3-1), suggesting that BcpAIOB-mediated inter-species CDI does occur. It is also notable that many, if not all, *bcpAI(O)B* genes are located on genomic islands. Several *Burkholderia* species have been shown to be naturally competent [29] and there is tremendous genomic diversity amongst *Burkholderia* strains, mediated in part by horizontal gene transfer [30]. These observations raise several questions. For example, is there a hierarchy of potency amongst the various BcpA proteins? What happens if bacteria with different *bcpAIOB* alleles come into contact? Or if a bacterium containing one allele encounters a bacterium containing two alleles? Is it advantageous for a bacterium to contain multiple alleles? If so, why is the greatest number of alleles identified in a single strain only three? Is there a cost associated with *bcpAIOB* alleles that limits the number that can be tolerated within a single cell? The question of how diversity amongst *bcpAIOB* alleles is generated is a perplexing one as nucleotide changes in the region encoding BcpA-CT can presumably be tolerated only if compensatory changes occur in *bcpI*, and vice versa. Finally, the most important question and one related to CDI in general but perhaps the most difficult to address experimentally: What is the true role of CDI in nature? Is it used for competitive exclusion or for the acquisition of DNA that can be used for nutrition, as a biofilm matrix, or a source of genetic diversity? Perhaps it serves all of these purposes – or none and plays a role that we cannot yet imagine. Because life in a community is the rule rather than the exception for most bacteria, understanding how and why CDI systems function will be broadly relevant.

While this manuscript was in revision, Nikolakakis *et al.* published a related characterization of CDI systems in *Burkholderia* [31]. Although these authors used different experimental approaches, their results are consistent with ours and support the conclusion that *bcpAIOB* genes encode CDI systems that can mediate interbacterial competition and that function in an allele-specific manner. Moreover, Nikolakakis *et al.* showed that the C-terminal domains of some BcpA proteins possess tRNase activity, similar to what has been demonstrated for the C-terminal domains of CdiA proteins in *E. coli* type CDI systems [31].

## Materials and Methods

### Strain construction and culture conditions

*Burkholderia thailandensis* E264 is an environmental isolate [14]. Plasmids were maintained in *E. coli* DH5α and DH5αλpir and mated into *B. thailandensis* using the donor *E. coli* strain RHO3 [32].

*B. thailandensis* was cultured in low salt Luria-Bertani medium (LSLB, 0.5% NaCl) (unless otherwise stated), M63 minimal medium (supplemented with 1 mM MgSO_4_, 0.2% glucose, and 0.4% glycerol) [29], or M9 minimal medium (supplemented with 2 mM MgSO_4_, 0.1 mM CaCl_2_, and 0.2% glucose) supplemented, as appropriate, with 35 µg/ml chloramphenicol, 250 µg/ml kanamycin, 20 µg/ml tetracycline, 50 µg/ml trimethoprim, 40 µg/ml X-gal, or 0.1% chlorophenol alanine. Overnight cultures were aerated for ∼18 h at 37°C to OD_600_ ∼7–9, centrifuged at 16,000× *g* for 1 min, washed and resuspended in fresh LSLB broth or sterile phosphate buffered saline (PBS) for subsequent use. *E. coli* strains were cultured in Luria-Bertani (LB) medium supplemented, as appropriate, with 100 µg/ml ampicillin, 35 µg/ml chloramphenicol, 50 µg/ml kanamycin, 20 µg/ml tetracycline, 50 µg/ml trimethoprim, or 200 µg/ml diaminopamillic acid (for RHO3 strains).

The *B. thailandensis* Δ*bcpO* and Δ*bcpB* strains were created by allelic exchange. Deletion constructs were created by PCR amplifying (PFU Ultra polymerase, Agilent) ∼500 nucleotides 5′ of *bcpO* and *bcpB* (including the first three codons of the gene) and the ∼500 nucleotides 3′ of *bcpO* and *bcpB* (including the last three codons of the gene) from E264 genomic DNA. DNA fragments were restriction digested and cloned into allelic exchange vector pSM112, which carries a *pheS* counter-selectable marker. The resulting plasmids, pMA11 and pMA12, were used to create the Δ*bcpO* and Δ*bcpB* strains, respectively.

The *B. thailandensis* Δ*bcpAIOB* strain was created by natural transformation as described [29]. Briefly, ∼800 nucleotides 5′ of *bcpA* (including the first three codons of the gene) and ∼800 nucleotides 3′ of *bcpB* (including the last three codons of the gene) were amplified from genomic E264 DNA and the gene encoding kanamycin resistance (plus ∼500 nucleotides to include the promoter) was amplified (PFU Ultra polymerase, Agilent) from pUC18miniTn7(Km). Overlap PCR was performed to construct a single DNA product in which the kanamycin resistance-encoding gene was flanked by the regions 5′ to *bcpA* and 3′ to *bcpB*, which was subsequently transformed into *B. thailandensis*.

The constitutively active strain (P_S12_-*bcpAIOB*) was constructed as follows. Approximately 200 nucleotides 5′ to the ribosomal S12 protein-encoding gene (containing the promoter, P_S12_) and ∼750 nucleotides 3′ of the *bcpA* translation start site were PCR amplified (PFU Ultra polymerase, Agilent) from genomic E264 DNA, joined by overlap PCR, and cloned into pEXKm5 [32] to create pECG22, which was then used for cointegration into the chromosome of E264.

Strains used for Western blot analysis were constructed as follows. A DNA fragment corresponding to ∼1000 nucleotides of internal *bcpA* sequence with an encoding HA-tag located after F2633 of BcpA was constructed by overlap PCR and cloned into pEXKm5 [32]. The resulting plasmid, pECG17, was used for allelic exchange with *B. thailandensis* E264, E264Δ*bcpO*, and E264Δ*bcpB* to generate the strains E264BcpA-HA, E264BcpA-HAΔ*bcpO*, and E264BcpA-HAΔ*bcpB*, respectively. pECG22 was then mated into these strains to yield E264BcpA-HA::pECG22, E264BcpA-HAΔ*bcpO*::pECG22, and E264BcpA-HAΔ*bcpB*::pECG22 such that P_S12_ would drive expression of an intact *bcpAIOB* locus in which the encoding BcpA protein contained an HA-tag located after F2633. Complementation of *bcpO* and *bcpB* is described below; strains yielded were: E264BcpA-HAΔ*bcpO*::*bcpO*::pECG22 and E264BcpA-HAΔ*bcpB*::*bcpB*::pECG22, respectively. The *bcpO* and *bcpB* genes were expressed from the constitutive promoter P_S12_.

Strains complemented with *bcpO*, *bcpI*, and *bcpB* were constructed using a Tn7 transposase as described [33]. Derivatives of pUC18T-mini-Tn7T-Gm were constructed in which the gentamycin resistance-encoding gene was replaced with a gene encoding kanamycin resistance (pUC18miniTn7(Km)), chloramphenicol resistance (pUC18miniTn7(Cm)), or tetracycline resistance (pUC18miniTn7(Tc)). Briefly, ∼200 nucleotides 5′ to the ribosomal S12 protein-encoding gene (containing the promoter, P_S12_) or 500 nucleotides 5′ to *bcpA* (containing the promoter, P*_bcpA_*) and *bcpO*, *bcpI*, or *bcpB* were amplified from genomic E264 DNA, restriction digested, and cloned into pUC18miniTn7(Km) or pUC18miniTn7(Tc) for insertion at the *att*Tn7 site on the chromosome.

*B. thailandensis* E264Cm^R^ and E264Km^R^ (microscopy and flow cytometery vector control) were constructed using pUC18miniTn7(Cm) and pUC18miniTn7(Km) to insert a gene encoding chloramphenicol or kanamycin resistance, respectively, on the chromosome at the *att*Tn7 site.

All *lacZ* and *gfp* reporter strains were created using the Tn7 transposase method as well. Briefly, ∼500 nucleotides 5′ to *bcpA* (P*_bcpA_*), ∼200 nucleotides corresponding to the predicted S12 promoter (P_S12_), or no DNA (P_neg_) were cloned 5′ to a promoterless *lacZ* gene and ∼500 nucleotides 5′ to *bcpA* (P*_bcpA_*) was cloned 5′ to a promoterless *gfp* gene and cloned into pUC18miniTn7(Km). Transposition into the chromosome of *B. thailandensis* at the *att*Tn7 site generated the P*_bcpA_*-*lacZ*, P_S12_-*lacZ*, P_neg_-*lacZ*, and P*_bcpA_*-*gfp* strains. The miniTn7-*kan*-*gfp* plasmid [34] was used to generate the *B. thailandensis* P_S12_-*gfp* reporter strain in a similar manner.

All strains constructed were verified by PCR and sequencing analysis (Eton BioScience).

### Bioinformatics

*Burkholderia*-type CDI-encoding loci were identified by using the predicted BcpB protein sequence from *B. pseudomallei* K96243 as a pBLAST query against the entire NCBI genome database. DNA sequences encoding predicted TPS system-like proteins were analyzed in Vector NTI Advance 11. A locus was considered to encode a *Burkholderia*-type CDI system if it was composed of a large ORF (∼3000 codons) followed immediately 3′ by a small ORF (∼100 codons), both of which were 5′ to the *tpsB* homolog. Alignments were performed in Vector NTI and then analyzed in Jalview 2.7 with Taylor residue coloring.

### Reverse transcriptase PCR

Total RNA was extracted from bacterial cells cultured overnight in LSLB broth using TRIzol Reagent (Invitrogen) according to the manufacturer's protocol. RNA was treated with 4 U of DNaseI (Ambion) for 30 min at 37°C. 2–5 µg of RNA was subsequently used for cDNA synthesis using SuperScript III First-Strand (Invitrogen) according to the manufacturer's protocol. RT-PCR was performed using GoTaq polymerase (Promega); PCR was also performed on E264 genomic DNA with identical primer sets as a control. PCR products were analyzed by 0.8% agarose gel electrophoresis and stained with ethidium bromide for visualization.

### β-galactosidase assay

Reporter strains were cultured overnight in LSLB at 37°C with or without aeration, at room temperature (∼25°C) with aeration, or in M63 or M9 minimal medium at 37°C with aeration. P_neg_-*lacZ* and P_S12_-*lacZ* were cultured in LSLB at 37°C with aeration. Heat shocked samples were obtained by incubating 500 µl aliquots of overnight LSLB (37°C, aerated) culture at 42°C for 5 min. Cultures were normalized to OD_600_ ∼0.2–0.5 and β-galactosidase activity was measured as described [35]. Two independent assays were performed in triplicate.

### Autoaggregation

Overnight cultures of strains in LSLB medium were diluted to OD_600_ = 0.2 into 2 ml of M63 minimal medium in glass tubes. Bacteria were cultured with aeration for ∼48 h at 37°C with or without 4 or 10 U DNaseI with 10× DNaseI buffer (Ambion).

### Immunoblotting

WT (E264BcpA-HA::pECG22), Δ*bcpO* (E264BcpA-HAΔ*bcpO*::pECG22), Δ*bcpB* (E264BcpA-HAΔ*bcpB*::pECG22), Δ*bcpO*::*bcpO* (E264BcpA-HAΔ*bcpO*::*bcpO*::pECG22), Δ*bcpB*::*bcpB* (E264BcpA-HAΔ*bcpB*::*bcpB*::pECG22), and no tag (E264::pECG22) strains were cultured overnight in LSLB supplemented with kanamycin. Cultures were washed twice in LSLB and diluted into fresh LSLB containing antibiotics. Bacteria were cultured overnight, pelleted, and resuspended in 2× SDS PAGE loading buffer to OD_600_ ∼50. Boiled samples were separated on a 5% SDS PAGE gel, transferred to nitrocellulose, and probed with mouse monoclonal anti-HA.11 antibody (Covance) at 1∶1,000 and goat anti-mouse IgG conjugated to IRDye680 (Odyssey) at 1∶15,000. Images were acquired on a Li-Cor (Odyssey) with software Odyssey v3.0.

### Intracellular toxicity

The last ∼350 codons of *bcpA* (including the Nx(E/Q)LYN motif) from *B. pseudomallei* strains K96243 and 1106A-2 were PCR amplified (with an added ATG at the 5′ end) from genomic DNA and cloned into pSCrhaB2 [36]. Plasmids carrying *bcpI* were cloned in a similar manner except the trimethoprim resistance gene of pSCrhaB2 was replaced with a kanamycin resistance gene to allow for selection of these plasmids in combination with the plasmids encoding BcpA-CTs. *B. thailandensis* containing the BcpA-CT-encoding plasmids alone or in combination with the BcpI-encoding plasmids were cultured overnight in LSLB supplemented with trimethoprim (strains with BcpA-CT-encoding plasmids) or trimethoprim and kanamycin (strains with BcpA-CT- and BcpI-encoding plasmids) and 0.2% glucose. Cultures were washed and diluted in fresh LSLB medium to OD_600_ 0.2–0.6 supplemented with antibiotics and 0.2% glucose or 0.2% rhamnose. Bacteria were cultured with aeration for 4 h at 37°C. Aliquots were taken at 0 h and 4 h, diluted in PBS, and plated on LSLB with antibiotic selection and 0.2% glucose to determine the cfu/ml of each strain. Two independent experiments were performed in triplicate.

### Microscopy

Confocal microscopy– Liquid cultured bacteria preparation; bacteria were cultured overnight in LSLB broth and M63 minimal medium and concentrated to OD_600_ ∼50 in PBS. Solid medium cultured bacteria preparation; bacteria in colony biofilms on agar were cut out from petri dishes (agar included) and placed on a glass slide. Cover slips were added to the top of the colony biofilms. Bacteria were imaged on a Ziess LSM5 Pascal Confocal Laser Scanning microscope, 63× objective with oil immersion. Macroscope– Colony biofilms were imaged with a Leica M420 macroscope, 0.5× objective, at 1, 2, and 4 days post inoculation. Live-imaging microscopy– 500 µl of LSLB agar was added to cover glass bottom dishes, Delta T, (Biotechs) and allowed to solidify. Bacteria were plated onto the agar and imaged on an Olympus IX81 inverted microscope at 20× objective.

### Flow cytometry

Bacteria were cultured overnight in LSLB broth and M63 minimal medium supplemented with kanamycin and diluted to OD_600_ ∼0.1 in PBS. Bacterial samples were analyzed on a Gallios Flow Cytometer (Beckman Coulter); bacteria were gated based on forward scatter and side scatter (∼1 um diameter and distinct from particles in the PBS only control (Figure S3)) for subsequent analysis. Data were analyzed with Kaluza 1.1 software.

### Competitions

Liquid– bacteria were cultured overnight, washed with PBS, and diluted to OD_600_ 0.2 in fresh LSLB medium. Strains were mixed at a 1∶1 ratio (final volume of 2 ml) without antibiotic selection and cultured with aeration for 24 h at 37°C. Aliquots were taken at 0 h and 24 h, diluted in PBS, and plated on LSLB with antibiotic selection to determine the cfu/ml of each strain in the competition. Two independent experiments were performed in triplicate.

Solid– bacteria were cultured overnight, washed with PBS, and diluted to OD_600_ 0.2 in LSLB. Strains were mixed at a 1∶1 ratio and 20 µl of culture was plated on solid LSLB (1.5% agar) without antibiotic selection. The culture inoculum was plated on LSLB with antibiotic selection to determine the ratio at 0 hours. Agar plates were stored at room temperature (∼25°C) for the duration of the experiment. Bacteria were picked from the colony biofilms with a sterile pipette tip, diluted in PBS, and plated on LSLB with antibiotic selection to determine the cfu of each strain in the competition at a particular dilution. The competitive index for each day was calculated as the ratio of wild type bacteria to mutant bacteria at time X hours divided by the ratio of wild type to mutant at time 0 hours. Two to three independent experiments were performed in triplicate.

## Supporting Information

Figure S1 Alignment of all predicted BcpA-CTs identified by bioinformatic analysis. The amino acid sequence of all predicted BcpA-CTs (50 aa N-terminal to the Nx(E/Q)LYN motif to the C-terminus of the protein) encoded by *Burkholderia*-type CDI loci were aligned in Vector NTI and analyzed in Jalview with Taylor residue coloring.
(PDF)

Figure S2 Evidence for lack of transcription polar effects in Δ*bcpO* and Δ*bcpB* strains. RT-PCR was performed on RNA extracted from Δ*bcpO* and Δ*bcpB* strains with primer sets annealing internal to *bcpB* (*bcpB* F+R) and *bcpO* (*bcpO* F+R), respectively. PCR was also performed on genomic E264 DNA as a control.
(TIF)

Figure S3 PBS control for flow cytometry analysis. Events collected in the PBS control for flow cytometry experiments were considered background (“Noise”) and excluded from further analysis.
(TIF)

Figure S4 Live-image microscopy of colony biofilms on agar. Colony biofilms were imaged through LSLB agar in a glass bottom dish at 20× objective in the center and edge at the indicated times. White arrows indicate individual bacteria, contacting bacteria, or dense bacteria. Black arrows indicate agar. Images represent authentic architecture of colony biofilms.
(TIF)

Figure S5 Confocal microscopy of colony biofilms on agar. Microscopy of E264P_S12_-*rfp* and E264P_S12_-*gfp* mixed at a 1∶1 ratio in the center (left column) and edge (right column) of colony biofilms at the indicated times.
(TIF)

Table S1 Predicted *Burkholderia*-type CDI system-encoding loci.
(PDF)

Table S2 Quantification of *bcpA*-*gfp* expression by flow cytometry.
(PDF)

Table S3 RT-PCR primer sequences.
(PDF)
