# Supplementary material for: The Burkholderia bcpAIOB Genes Define Unique Classes of Two-Partner Secretion and Contact Dependent Growth Inhibition Systems
Source: PLoS Genet. 2012 Aug 9;8(8):e1002877. doi: 10.1371/journal.pgen.1002877 (PMC3415462; doi:10.1371/journal.pgen.1002877)
Supplement: Figure S1 — Alignment of all predicted BcpA-CTs identified by bioinformatic analysis. The amino acid sequence of all predicted BcpA-CTs (50 aa N-terminal to the Nx(E/Q)LYN motif to the C-terminus of the protein) encoded by Burkholderia-type CDI loci were aligned in Vector NTI and analyzed in Jalview with Taylor residue coloring. (PDF) [file pgen.1002877.s001.pdf]

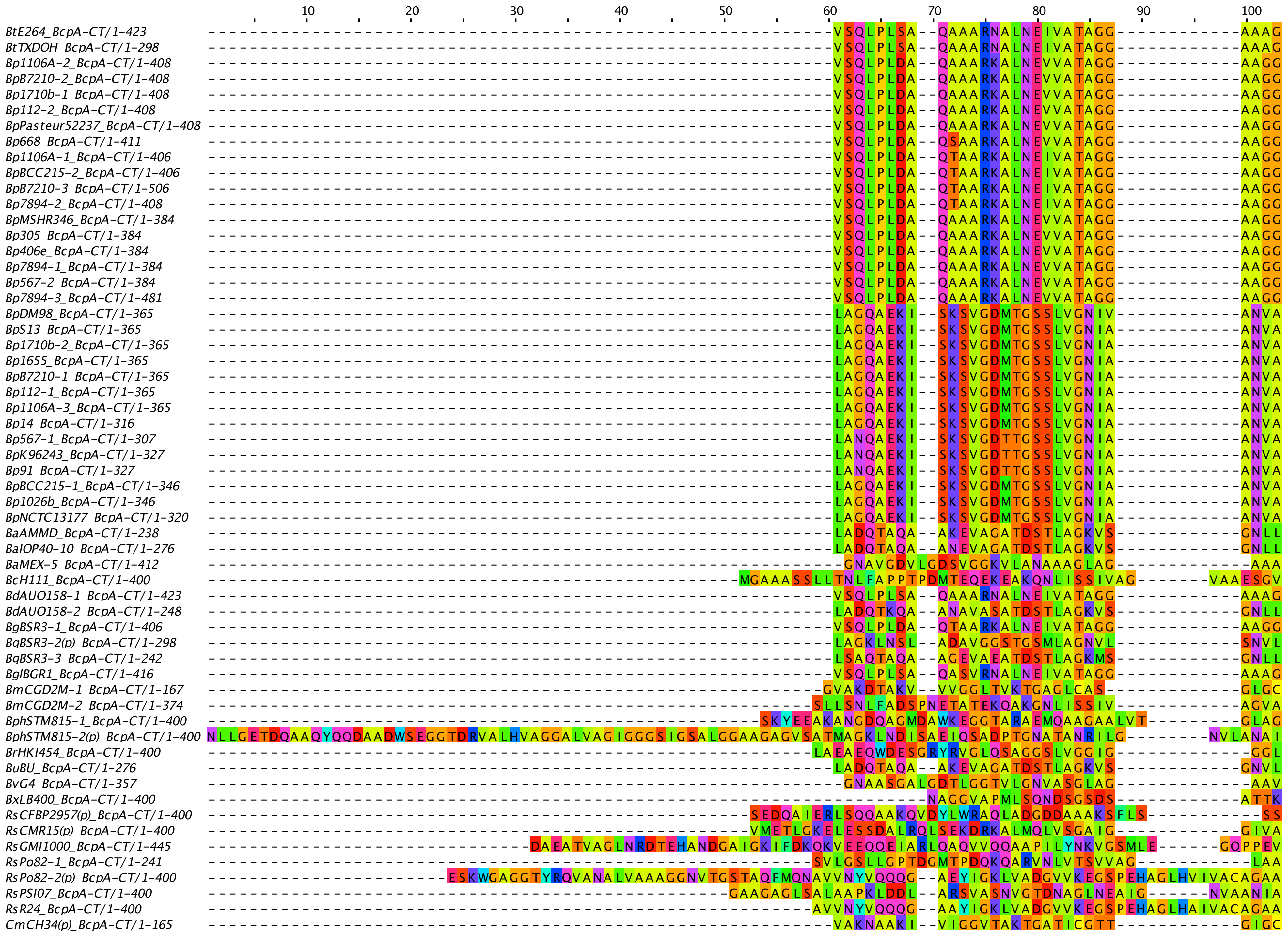
10 20 30 40 50 60 70 80 90 100
BtE264_BcpA-CT/1-423
BtTXDOH_BcpA-CT/1-298
Bp1106A-2_BcpA-CT/1-408
BpB7210-2_BcpA-CT/1-408
Bp1710b-1_BcpA-CT/1-408
Bp112-2_BcpA-CT/1-408
BpPasteur52237_BcpA-CT/1-408
Bp668_BcpA-CT/1-411
Bp1106A-1_BcpA-CT/1-406
BpBCC215-2_BcpA-CT/1-406
BpB7210-3_BcpA-CT/1-506
Bp7894-2_BcpA-CT/1-408
BpMSHR346_BcpA-CT/1-384
Bp305_BcpA-CT/1-384
Bp406e_BcpA-CT/1-384
Bp7894-1_BcpA-CT/1-384
Bp567-2_BcpA-CT/1-384
Bp7894-3_BcpA-CT/1-481
BpDM98_BcpA-CT/1-365
BpS13_BcpA-CT/1-365
Bp1710b-2_BcpA-CT/1-365
Bp1655_BcpA-CT/1-365
BpB7210-1_BcpA-CT/1-365
Bp112-1_BcpA-CT/1-365
Bp1106A-3_BcpA-CT/1-365
Bp14_BcpA-CT/1-316
Bp567-1_BcpA-CT/1-307
BpK96243_BcpA-CT/1-327
Bp91_BcpA-CT/1-327
BpBCC215-1_BcpA-CT/1-346
Bp1026b_BcpA-CT/1-346
BpNCTC13177_BcpA-CT/1-320
BaAMMD_BcpA-CT/1-238
BaIOP40-10_BcpA-CT/1-276
BaMEX-5_BcpA-CT/1-412
BcH111_BcpA-CT/1-400
BdAUO158-1_BcpA-CT/1-423
BdAUO158-2_BcpA-CT/1-248
BgBSR3-1_BcpA-CT/1-406
BgBSR3-2(p)_BcpA-CT/1-298
BgBSR3-3_BcpA-CT/1-242
BglBGR1_BcpA-CT/1-416
BmCGD2M-1_BcpA-CT/1-167
BmCGD2M-2_BcpA-CT/1-374
BphSTM815-1_BcpA-CT/1-400
BphSTM815-2(p)_BcpA-CT/1-400
BrHKI454_BcpA-CT/1-400
BuBU_BcpA-CT/1-276
BvG4_BcpA-CT/1-357
BxLB400_BcpA-CT/1-400
RsCFBP2957(p)_BcpA-CT/1-400
RsCMR15(p)_BcpA-CT/1-400
RsGMI1000_BcpA-CT/1-445
RsPo82-1_BcpA-CT/1-241
RsPo82-2(p)_BcpA-CT/1-400
RsPSI07_BcpA-CT/1-400
RsR24_BcpA-CT/1-400
CmCH34(p)_BcpA-CT/1-165

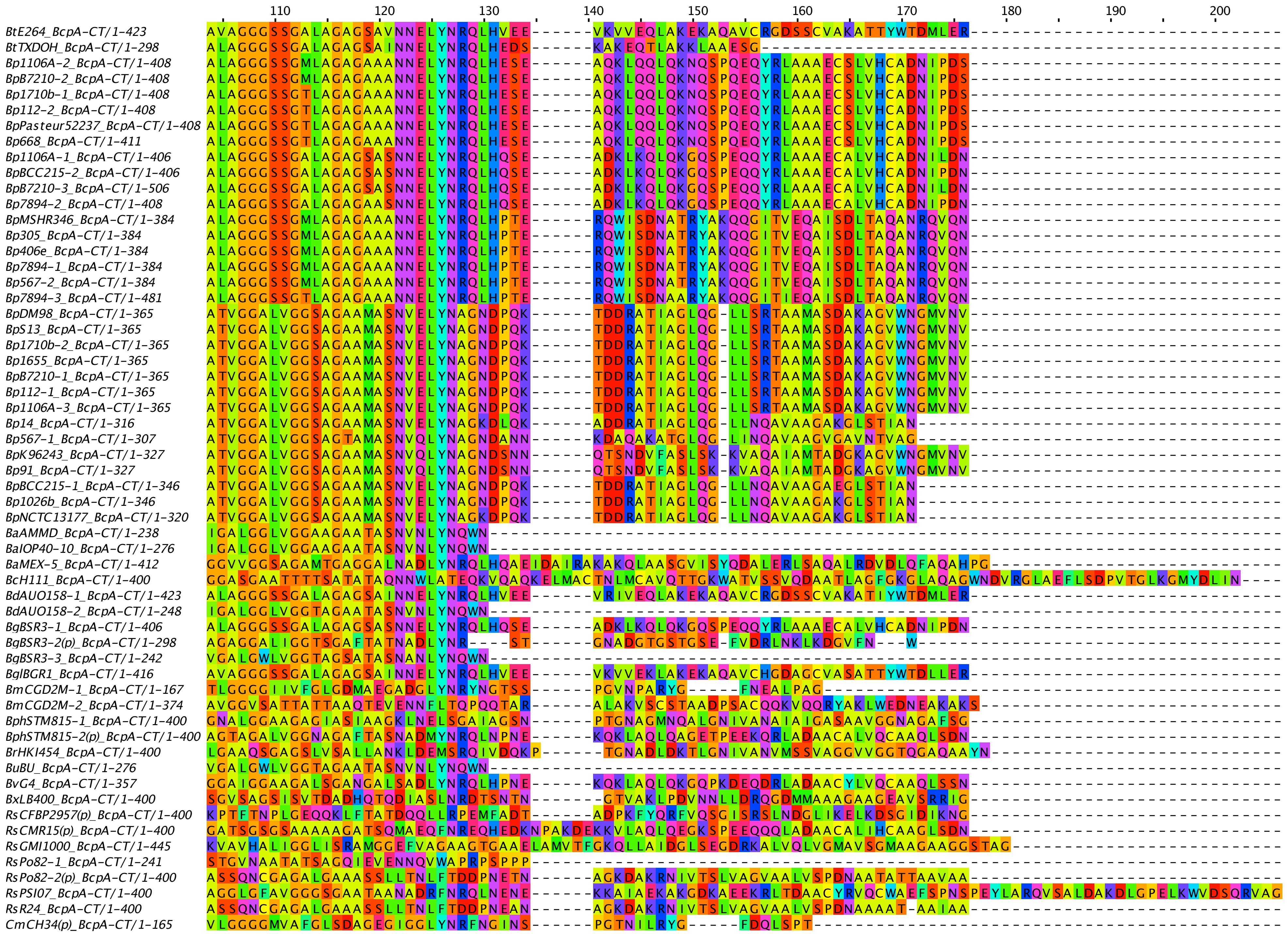
110
120
130
140
150
160
170
180
190
200
BtE264_BcpA-CT/1-423
BtTXDOH_BcpA-CT/1-298
Bp1106A-2_BcpA-CT/1-408
BpB7210-2_BcpA-CT/1-408
Bp1710b-1_BcpA-CT/1-408
Bp112-2_BcpA-CT/1-408
BpPasteur52237_BcpA-CT/1-408
Bp668_BcpA-CT/1-411
Bp1106A-1_BcpA-CT/1-406
BpBCC215-2_BcpA-CT/1-406
BpB7210-3_BcpA-CT/1-506
Bp7894-2_BcpA-CT/1-408
BpMSHR346_BcpA-CT/1-384
Bp305_BcpA-CT/1-384
Bp406e_BcpA-CT/1-384
Bp7894-1_BcpA-CT/1-384
Bp567-2_BcpA-CT/1-384
Bp7894-3_BcpA-CT/1-481
BpDM98_BcpA-CT/1-365
BpS13_BcpA-CT/1-365
Bp1710b-2_BcpA-CT/1-365
Bp1655_BcpA-CT/1-365
BpB7210-1_BcpA-CT/1-365
Bp112-1_BcpA-CT/1-365
Bp1106A-3_BcpA-CT/1-365
Bp14_BcpA-CT/1-316
Bp567-1_BcpA-CT/1-307
BpK96243_BcpA-CT/1-327
Bp91_BcpA-CT/1-327
BpBCC215-1_BcpA-CT/1-346
Bp1026b_BcpA-CT/1-346
BpNCTC13177_BcpA-CT/1-320
BaAMMD_BcpA-CT/1-238
BaIOP40-10_BcpA-CT/1-276
BaMEX-5_BcpA-CT/1-412
BcH111_BcpA-CT/1-400
BdAUO158-1_BcpA-CT/1-423
BdAUO158-2_BcpA-CT/1-248
BgBSR3-1_BcpA-CT/1-406
BgBSR3-2(p)_BcpA-CT/1-298
BgBSR3-3_BcpA-CT/1-242
BglBGR1_BcpA-CT/1-416
BmCGD2M-1_BcpA-CT/1-167
BmCGD2M-2_BcpA-CT/1-374
BphSTM815-1_BcpA-CT/1-400
BphSTM815-2(p)_BcpA-CT/1-400
BrHKI454_BcpA-CT/1-400
BuBU_BcpA-CT/1-276
BvG4_BcpA-CT/1-357
BxLB400_BcpA-CT/1-400
RsCFBP2957(p)_BcpA-CT/1-400
RsCMR15(p)_BcpA-CT/1-400
RsGMI1000_BcpA-CT/1-445
RsPo82-1_BcpA-CT/1-241
RsPo82-2(p)_BcpA-CT/1-400
RsPSI07_BcpA-CT/1-400
RsR24_BcpA-CT/1-400
CmCH34(p)_BcpA-CT/1-165

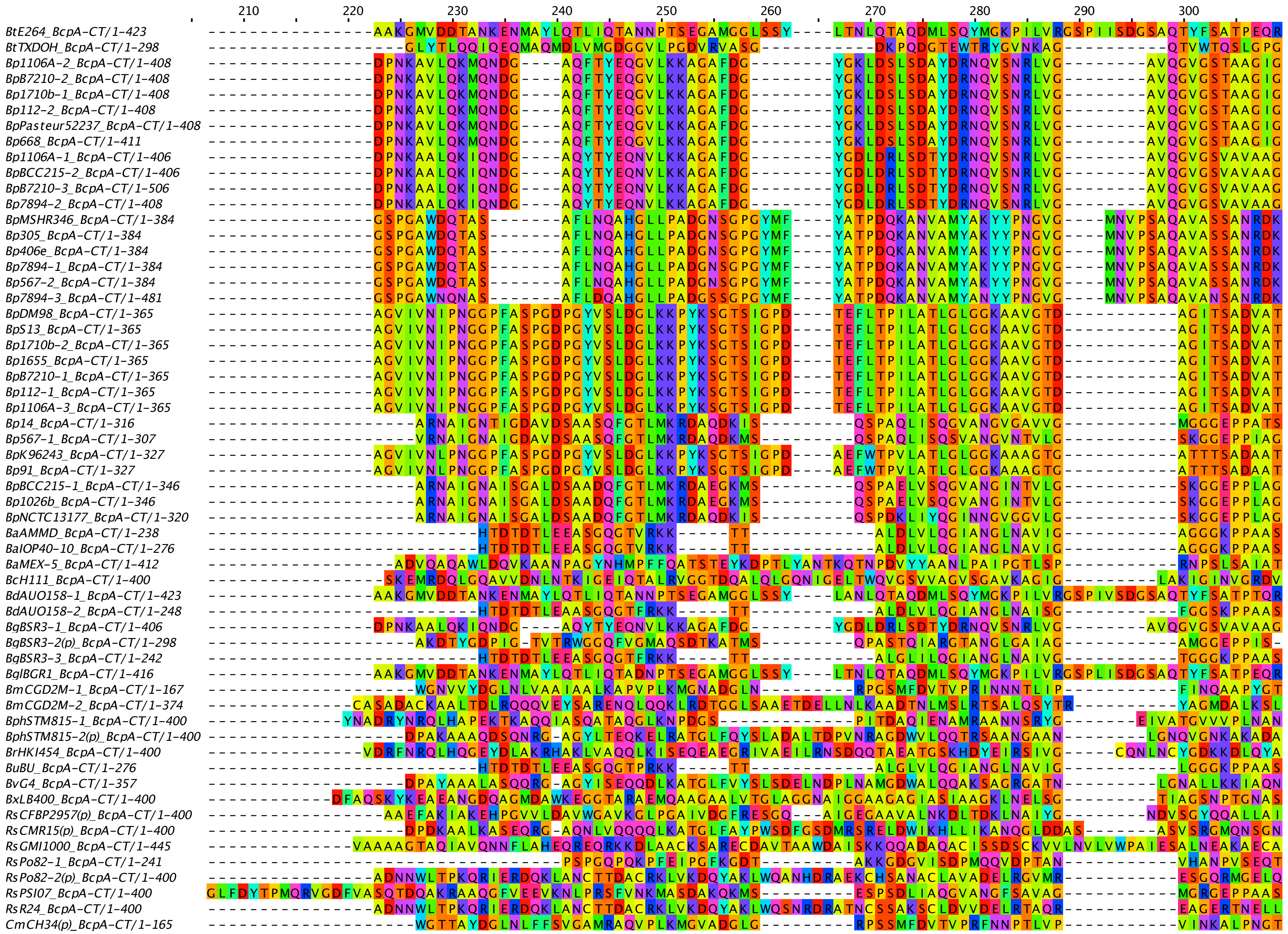

210
220
230
240
250
260
270
280
290
300
BtE264_BcpA-CT/1-423
BtTXDOH_BcpA-CT/1-298
Bp1106A-2_BcpA-CT/1-408
BpB7210-2_BcpA-CT/1-408
Bp1710b-1_BcpA-CT/1-408
Bp112-2_BcpA-CT/1-408
BpPasteur52237_BcpA-CT/1-408
Bp668_BcpA-CT/1-411
Bp1106A-1_BcpA-CT/1-406
BpBCC215-2_BcpA-CT/1-406
BpB7210-3_BcpA-CT/1-506
Bp7894-2_BcpA-CT/1-408
BpMSHR346_BcpA-CT/1-384
Bp305_BcpA-CT/1-384
Bp406e_BcpA-CT/1-384
Bp7894-1_BcpA-CT/1-384
Bp567-2_BcpA-CT/1-384
Bp7894-3_BcpA-CT/1-481
BpDM98_BcpA-CT/1-365
BpS13_BcpA-CT/1-365
Bp1710b-2_BcpA-CT/1-365
Bp1655_BcpA-CT/1-365
BpB7210-1_BcpA-CT/1-365
Bp112-1_BcpA-CT/1-365
Bp1106A-3_BcpA-CT/1-365
Bp14_BcpA-CT/1-316
Bp567-1_BcpA-CT/1-307
BpK96243_BcpA-CT/1-327
Bp91_BcpA-CT/1-327
BpBCC215-1_BcpA-CT/1-346
Bp1026b_BcpA-CT/1-346
BpNCTC13177_BcpA-CT/1-320
BaAMMD_BcpA-CT/1-238
BaIOP40-10_BcpA-CT/1-276
BaMEX-5_BcpA-CT/1-412
BcH111_BcpA-CT/1-400
BdAUO158-1_BcpA-CT/1-423
BdAUO158-2_BcpA-CT/1-248
BgBSR3-1_BcpA-CT/1-406
BgBSR3-2(p)_BcpA-CT/1-298
BgBSR3-3_BcpA-CT/1-242
BglBGR1_BcpA-CT/1-416
BmCGD2M-1_BcpA-CT/1-167
BmCGD2M-2_BcpA-CT/1-374
BphSTM815-1_BcpA-CT/1-400
BphSTM815-2(p)_BcpA-CT/1-400
BrHKI454_BcpA-CT/1-400
BuBU_BcpA-CT/1-276
BvG4_BcpA-CT/1-357
BxLB400_BcpA-CT/1-400
RsCFBP2957(p)_BcpA-CT/1-400
RsCMR15(p)_BcpA-CT/1-400
RsGMI1000_BcpA-CT/1-445
RsPo82-1_BcpA-CT/1-241
RsPo82-2(p)_BcpA-CT/1-400
RsPSI07_BcpA-CT/1-400
RsR24_BcpA-CT/1-400
CmCH34(p)_BcpA-CT/1-165

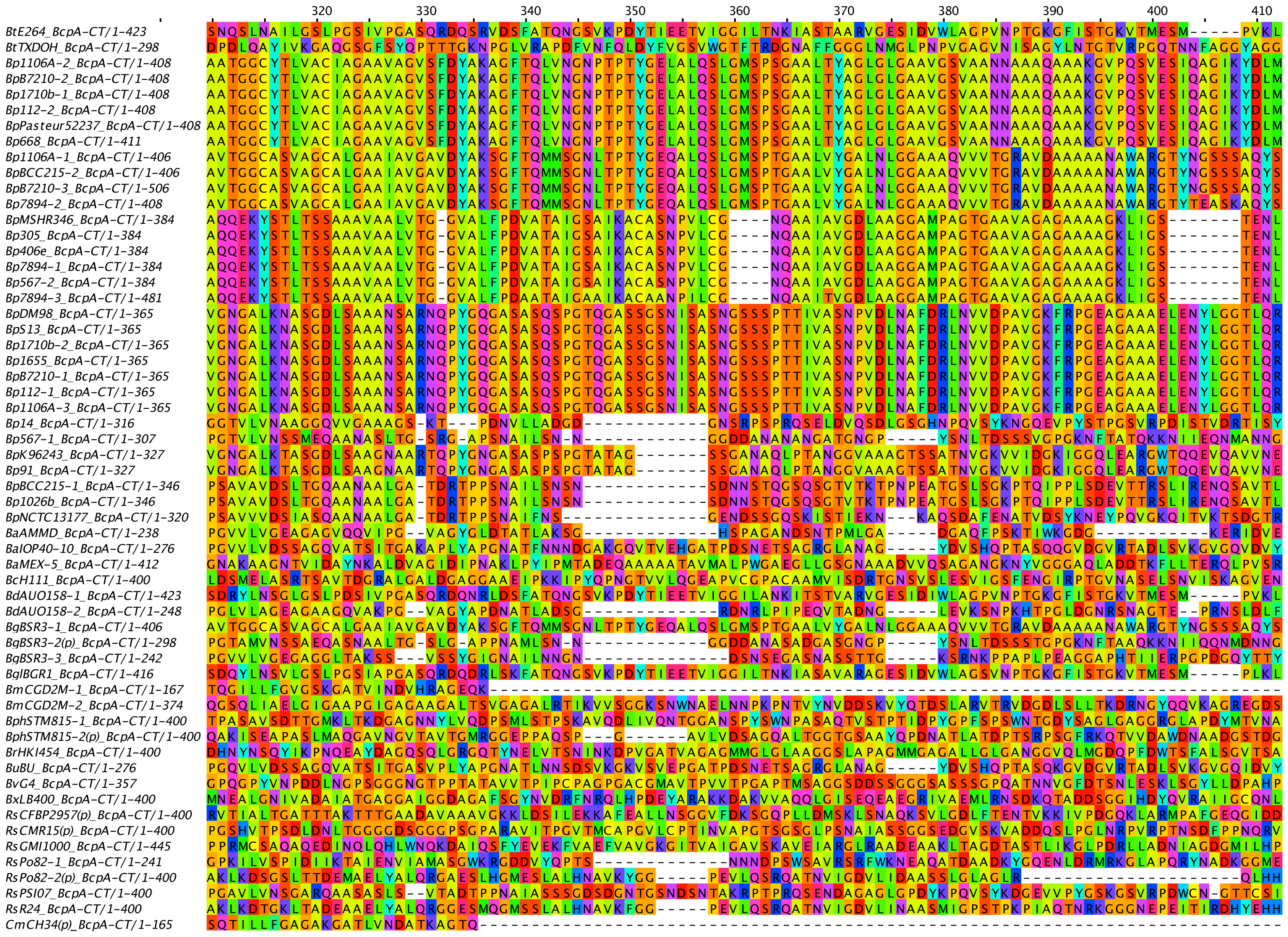

320
330
340
350
360
370
380
390
400
410
BtE264_BcpA-CT/1-423
BtTXDOH_BcpA-CT/1-298
Bp1106A-2_BcpA-CT/1-408
BpB7210-2_BcpA-CT/1-408
Bp1710b-1_BcpA-CT/1-408
Bp112-2_BcpA-CT/1-408
BpPasteur52237_BcpA-CT/1-408
Bp668_BcpA-CT/1-411
Bp1106A-1_BcpA-CT/1-406
BpBCC215-2_BcpA-CT/1-406
BpB7210-3_BcpA-CT/1-506
Bp7894-2_BcpA-CT/1-408
BpMSHR346_BcpA-CT/1-384
Bp305_BcpA-CT/1-384
Bp406e_BcpA-CT/1-384
Bp7894-1_BcpA-CT/1-384
Bp567-2_BcpA-CT/1-384
Bp7894-3_BcpA-CT/1-481
BpDM98_BcpA-CT/1-365
BpS13_BcpA-CT/1-365
Bp1710b-2_BcpA-CT/1-365
Bp1655_BcpA-CT/1-365
BpB7210-1_BcpA-CT/1-365
Bp112-1_BcpA-CT/1-365
Bp1106A-3_BcpA-CT/1-365
Bp14_BcpA-CT/1-316
Bp567-1_BcpA-CT/1-307
BpK96243_BcpA-CT/1-327
Bp91_BcpA-CT/1-327
BpBCC215-1_BcpA-CT/1-346
Bp1026b_BcpA-CT/1-346
BpNCTC13177_BcpA-CT/1-320
BaAMMD_BcpA-CT/1-238
BaIOP40-10_BcpA-CT/1-276
BaMEX-5_BcpA-CT/1-412
BcH111_BcpA-CT/1-400
BdAUO158-1_BcpA-CT/1-423
BdAUO158-2_BcpA-CT/1-248
BgBSR3-1_BcpA-CT/1-406
BgBSR3-2(p)_BcpA-CT/1-298
BgBSR3-3_BcpA-CT/1-242
BglBGR1_BcpA-CT/1-416
BmCGD2M-1_BcpA-CT/1-167
BmCGD2M-2_BcpA-CT/1-374
BphSTM815-1_BcpA-CT/1-400
BphSTM815-2(p)_BcpA-CT/1-400
BrHKI454_BcpA-CT/1-400
BuBU_BcpA-CT/1-276
BvG4_BcpA-CT/1-357
BxLB400_BcpA-CT/1-400
RsCFBP2957(p)_BcpA-CT/1-400
RsCMR15(p)_BcpA-CT/1-400
RsGMI1000_BcpA-CT/1-445
RsPo82-1_BcpA-CT/1-241
RsPo82-2(p)_BcpA-CT/1-400
RsPSI07_BcpA-CT/1-400
RsR24_BcpA-CT/1-400
CmCH34(p)_BcpA-CT/1-165

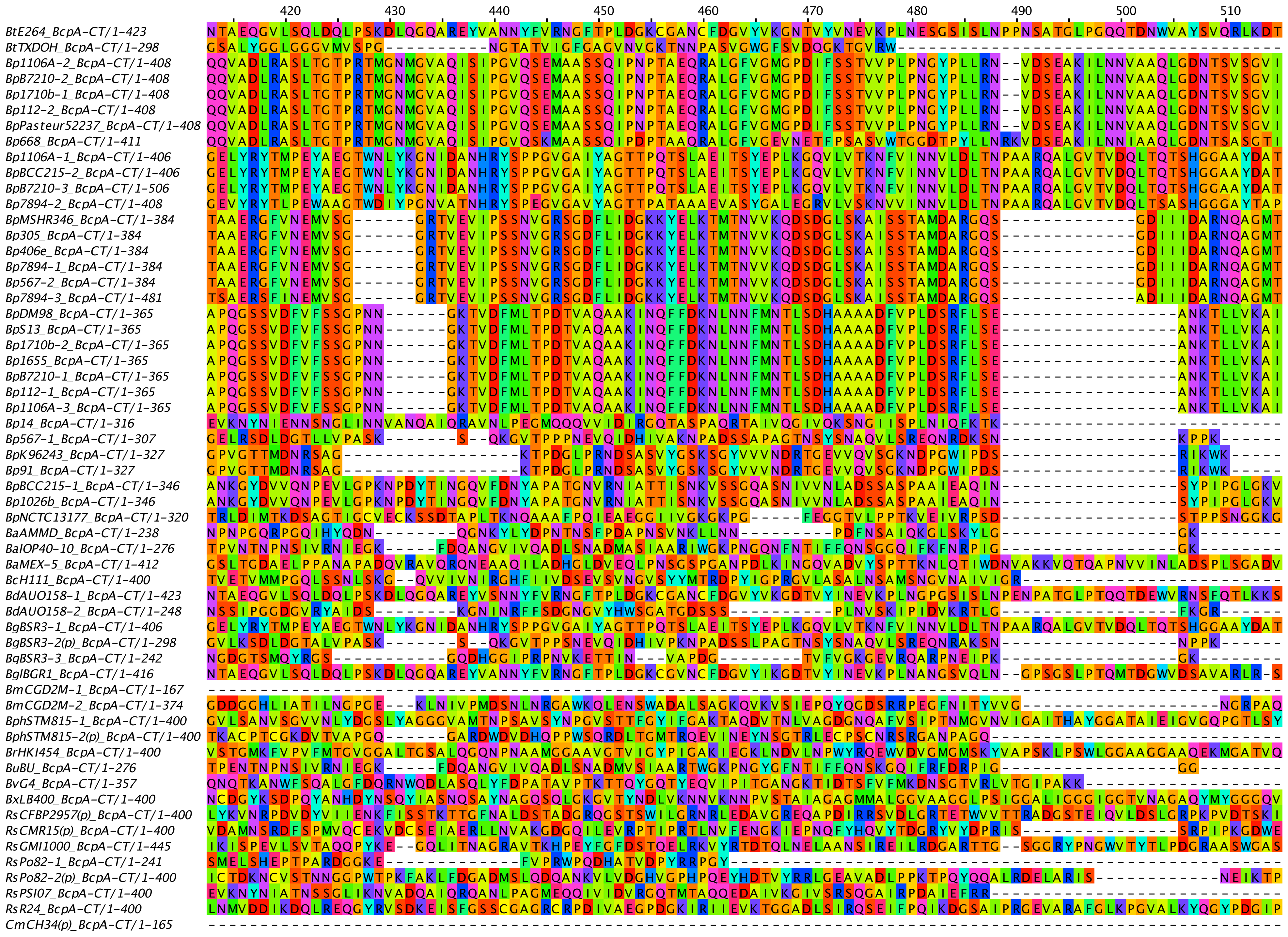
420
430
440
450
460
470
480
490
500
510
BtE264_BcpA-CT/1-423
BtTXDOH_BcpA-CT/1-298
Bp1106A-2_BcpA-CT/1-408
BpB7210-2_BcpA-CT/1-408
Bp1710b-1_BcpA-CT/1-408
Bp112-2_BcpA-CT/1-408
BpPasteur52237_BcpA-CT/1-408
Bp668_BcpA-CT/1-411
Bp1106A-1_BcpA-CT/1-406
BpBCC215-2_BcpA-CT/1-406
BpB7210-3_BcpA-CT/1-506
Bp7894-2_BcpA-CT/1-408
BpMSHR346_BcpA-CT/1-384
Bp305_BcpA-CT/1-384
Bp406e_BcpA-CT/1-384
Bp7894-1_BcpA-CT/1-384
Bp567-2_BcpA-CT/1-384
Bp7894-3_BcpA-CT/1-481
BpDM98_BcpA-CT/1-365
BpS13_BcpA-CT/1-365
Bp1710b-2_BcpA-CT/1-365
Bp1655_BcpA-CT/1-365
BpB7210-1_BcpA-CT/1-365
Bp112-1_BcpA-CT/1-365
Bp1106A-3_BcpA-CT/1-365
Bp14_BcpA-CT/1-316
Bp567-1_BcpA-CT/1-307
BpK96243_BcpA-CT/1-327
Bp91_BcpA-CT/1-327
BpBCC215-1_BcpA-CT/1-346
Bp1026b_BcpA-CT/1-346
BpNCTC13177_BcpA-CT/1-320
BaAMMD_BcpA-CT/1-238
BaIOP40-10_BcpA-CT/1-276
BaMEX-5_BcpA-CT/1-412
BcH111_BcpA-CT/1-400
BdAUO158-1_BcpA-CT/1-423
BdAUO158-2_BcpA-CT/1-248
BgBSR3-1_BcpA-CT/1-406
BgBSR3-2(p)_BcpA-CT/1-298
BgBSR3-3_BcpA-CT/1-242
BglBGR1_BcpA-CT/1-416
BmCGD2M-1_BcpA-CT/1-167
BmCGD2M-2_BcpA-CT/1-374
BphSTM815-1_BcpA-CT/1-400
BphSTM815-2(p)_BcpA-CT/1-400
BrHKI454_BcpA-CT/1-400
BuBU_BcpA-CT/1-276
BvG4_BcpA-CT/1-357
BxLB400_BcpA-CT/1-400
RsCFBP2957(p)_BcpA-CT/1-400
RsCMR15(p)_BcpA-CT/1-400
RsGMI1000_BcpA-CT/1-445
RsPo82-1_BcpA-CT/1-241
RsPo82-2(p)_BcpA-CT/1-400
RsPSI07_BcpA-CT/1-400
RsR24_BcpA-CT/1-400
CmCH34(p)_BcpA-CT/1-165

Sequence	520 – 610
BtE264_BcpA-CT/1-423	GDPQLIKTAEVVEQAFRNGNLVKTVSGVNSNGMVVVKVPRNTP
BtTXDOH_BcpA-CT/1-298	
Bp1106A-2_BcpA-CT/1-408	NLFTERPPCTSCSNVIQQFQNKYPNIKINVMDSNGVLKPSK
BpB7210-2_BcpA-CT/1-408	NLFTERPPCTSCSNVIQQFQNKYPNIKINVMDSNGVLKPSK
Bp1710b-1_BcpA-CT/1-408	NLFTERPPCTSCSNVIQQFQNKYPNIKINVMDSNGVLKPSK
Bp112-2_BcpA-CT/1-408	NLFTERPPCTSCSNVIQQFQNKYPNIKINVMDSNGVLKPSK
BpPasteur52237_BcpA-CT/1-408	NLFTERPPCTSCSNVIQQFQNKYPNIKINVMDSNGVLKPSK
Bp668_BcpA-CT/1-411	NLFTERPPCESCSNTIIKFQEKYPNIKINVMDSNGVIRPSKR
Bp1106A-1_BcpA-CT/1-406	QAISTWAREQGYQAILAPSAQLPGGVNLISFKSLGKQ
BpBCC215-2_BcpA-CT/1-406	QAISTWAREQGYQAILAPSAQLPGGVNLISFKSLGKQ
BpB7210-3_BcpA-CT/1-506	QAISTWAREQGYQAILAPSAQLPGGVNLISFKSLGNSNMEDIPEGWGKFVDALRPSWEENDVTKELSNLVRDPEIAVAIWGCLKEGAITWLDYKAPILKKRKP
Bp7894-2_BcpA-CT/1-408	QAISVWAREQGYQAILAPSAQNAGGVNLISFKPLGEGRK
BpMSHR346_BcpA-CT/1-384	PEIAQRGIGRAYSRDTDTGAKIQSITVITPQGTVYVPRLPK
Bp305_BcpA-CT/1-384	PEIAQRGIGRAYSRDTDTGAKIQSITVITPQGTVYVPRLPK
Bp406e_BcpA-CT/1-384	PEIAQRGIGRAYSRDTDTGAKIQSITVITPQGTVYVPRLPK
Bp7894-1_BcpA-CT/1-384	PEIAQRGIGRAYSRDTDTGAKIQSITVITPQGTVYVPRLPK
Bp567-2_BcpA-CT/1-384	PEIAQRGIGRAYSRDTDTGAKIQSITVITPQGTVYVPRLPK
Bp7894-3_BcpA-CT/1-481	PEIATRGISRALSRDSGSGEKNSKHYHYYFTGDGVRPEVAEMIAMINGYPTAEELRNRILRQLEWRGPTTEVASVWRGYLTALLEWGLLEISDHDALASLLPV
BpDM98_BcpA-CT/1-365	GNLPQKLQAKIILIK
BpS13_BcpA-CT/1-365	GNLPQKLQAKIILIK
Bp1710b-2_BcpA-CT/1-365	GNLPQKLQAKIILIK
Bp1655_BcpA-CT/1-365	GNLPQKLQAKIILIK
BpB7210-1_BcpA-CT/1-365	GNLPQKLQAKIILIK
Bp112-1_BcpA-CT/1-365	GNLPQKLQAKIILIK
Bp1106A-3_BcpA-CT/1-365	GNLPQKLQAKIILIK
Bp14_BcpA-CT/1-316	
Bp567-1_BcpA-CT/1-307	
BpK96243_BcpA-CT/1-327	
Bp91_BcpA-CT/1-327	
BpBCC215-1_BcpA-CT/1-346	IVIDKLGNITIIKPKGN
Bp1026b_BcpA-CT/1-346	IVIDKLGNITIIKPKGN
BpNCTC13177_BcpA-CT/1-320	K
BaAMMD_BcpA-CT/1-238	
BaIOP40-10_BcpA-CT/1-276	
BaMEX-5_BcpA-CT/1-412	AQFLQRNPVAGMNTVTVIKNGAVVVVGR
BcH111_BcpA-CT/1-400	
BdAUO158-1_BcpA-CT/1-423	KDPDLVKTGSIIEKAFEEGKLVKLVSGVDSNGMVIVKIPRGSK
BdAUO158-2_BcpA-CT/1-248	
BgBSR3-1_BcpA-CT/1-406	QAISTWAREQGYQAILAPSAQLPGGVNLISFKSLGKQ
BgBSR3-2(p)_BcpA-CT/1-298	
BgBSR3-3_BcpA-CT/1-242	
BglBGR1_BcpA-CT/1-416	GDANQRATADLIQKAIDSGKLVKIVTGVNSNGATLVKIK
BmCGD2M-1_BcpA-CT/1-167	
BmCGD2M-2_BcpA-CT/1-374	QYFRNSPGGR
BphSTM815-1_BcpA-CT/1-400	GIVPWSHTTQVTGQSK
BphSTM815-2(p)_BcpA-CT/1-400	
BrHKI454_BcpA-CT/1-400	NKLDEEAAKK
BuBU_BcpA-CT/1-276	
BvG4_BcpA-CT/1-357	
BxLB400_BcpA-CT/1-400	SVVDVAIAGLTGAITYGTSLLPGL
RsCFBP2957(p)_BcpA-CT/1-400	LLPGSNAVGAKL
RsCMR15(p)_BcpA-CT/1-400	QHIRNINPGGVVISNKPQGLH
RsGMI1000_BcpA-CT/1-445	GDFIGFRGISK
RsPo82-1_BcpA-CT/1-241	
RsPo82-2(p)_BcpA-CT/1-400	GTLLNKLVTKQ
RsPSI07_BcpA-CT/1-400	
RsR24_BcpA-CT/1-400	VEIMNFPGAKNERK
CmCH34(p)_BcpA-CT/1-165	

```
                                   620       630       640       650
BtE264_BcpA-CT/1-423               -----------------------------------
BtTXDOH_BcpA-CT/1-298              -----------------------------------
Bp1106A-2_BcpA-CT/1-408            -----------------------------------
BpB7210-2_BcpA-CT/1-408            -----------------------------------
Bp1710b-1_BcpA-CT/1-408            -----------------------------------
Bp112-2_BcpA-CT/1-408              -----------------------------------
BpPasteur52237_BcpA-CT/1-408       -----------------------------------
Bp668_BcpA-CT/1-411                -----------------------------------
Bp1106A-1_BcpA-CT/1-406            -----------------------------------
BpBCC215-2_BcpA-CT/1-406           -----------------------------------
BpB7210-3_BcpA-CT/1-506            IDLLKTEKGRGEIRWMLISNPWWSGMGERSKKSE-
Bp7894-2_BcpA-CT/1-408             -----------------------------------
BpMSHR346_BcpA-CT/1-384            -----------------------------------
Bp305_BcpA-CT/1-384                -----------------------------------
Bp406e_BcpA-CT/1-384               -----------------------------------
Bp7894-1_BcpA-CT/1-384             -----------------------------------
Bp567-2_BcpA-CT/1-384              -----------------------------------
Bp7894-3_BcpA-CT/1-481             KGVKEVVELSADEPLDKESDAYIDEQMRLDEDKWK
BpDM98_BcpA-CT/1-365               -----------------------------------
BpS13_BcpA-CT/1-365                -----------------------------------
Bp1710b-2_BcpA-CT/1-365            -----------------------------------
Bp1655_BcpA-CT/1-365               -----------------------------------
BpB7210-1_BcpA-CT/1-365            -----------------------------------
Bp112-1_BcpA-CT/1-365              -----------------------------------
Bp1106A-3_BcpA-CT/1-365            -----------------------------------
Bp14_BcpA-CT/1-316                 -----------------------------------
Bp567-1_BcpA-CT/1-307              -----------------------------------
BpK96243_BcpA-CT/1-327             -----------------------------------
Bp91_BcpA-CT/1-327                 -----------------------------------
BpBCC215-1_BcpA-CT/1-346           -----------------------------------
Bp1026b_BcpA-CT/1-346              -----------------------------------
BpNCTC13177_BcpA-CT/1-320          -----------------------------------
BaAMMD_BcpA-CT/1-238               -----------------------------------
BaIOP40-10_BcpA-CT/1-276           -----------------------------------
BaMEX-5_BcpA-CT/1-412              -----------------------------------
BcH111_BcpA-CT/1-400               -----------------------------------
BdAUO158-1_BcpA-CT/1-423           -----------------------------------
BdAUO158-2_BcpA-CT/1-248           -----------------------------------
BgBSR3-1_BcpA-CT/1-406             -----------------------------------
BgBSR3-2(p)_BcpA-CT/1-298          -----------------------------------
BgBSR3-3_BcpA-CT/1-242             -----------------------------------
BglBGR1_BcpA-CT/1-416              -----------------------------------
BmCGD2M-1_BcpA-CT/1-167            -----------------------------------
BmCGD2M-2_BcpA-CT/1-374            -----------------------------------
BphSTM815-1_BcpA-CT/1-400          -----------------------------------
BphSTM815-2(p)_BcpA-CT/1-400       -----------------------------------
BrHKI454_BcpA-CT/1-400             -----------------------------------
BuBU_BcpA-CT/1-276                 -----------------------------------
BvG4_BcpA-CT/1-357                 -----------------------------------
BxLB400_BcpA-CT/1-400              -----------------------------------
RsCFBP2957(p)_BcpA-CT/1-400        -----------------------------------
RsCMR15(p)_BcpA-CT/1-400           -----------------------------------
RsGMI1000_BcpA-CT/1-445            -----------------------------------
RsPo82-1_BcpA-CT/1-241             -----------------------------------
RsPo82-2(p)_BcpA-CT/1-400          -----------------------------------
RsPSI07_BcpA-CT/1-400              -----------------------------------
RsR24_BcpA-CT/1-400                -----------------------------------
CmCH34(p)_BcpA-CT/1-165            -----------------------------------
```
