# Supplementary figures and images for: The Burkholderia bcpAIOB Genes Define Unique Classes of Two-Partner Secretion and Contact Dependent Growth Inhibition Systems
Source: PLoS Genet. 2012 Aug 9;8(8):e1002877. doi: 10.1371/journal.pgen.1002877 (PMC3415462; doi:10.1371/journal.pgen.1002877)

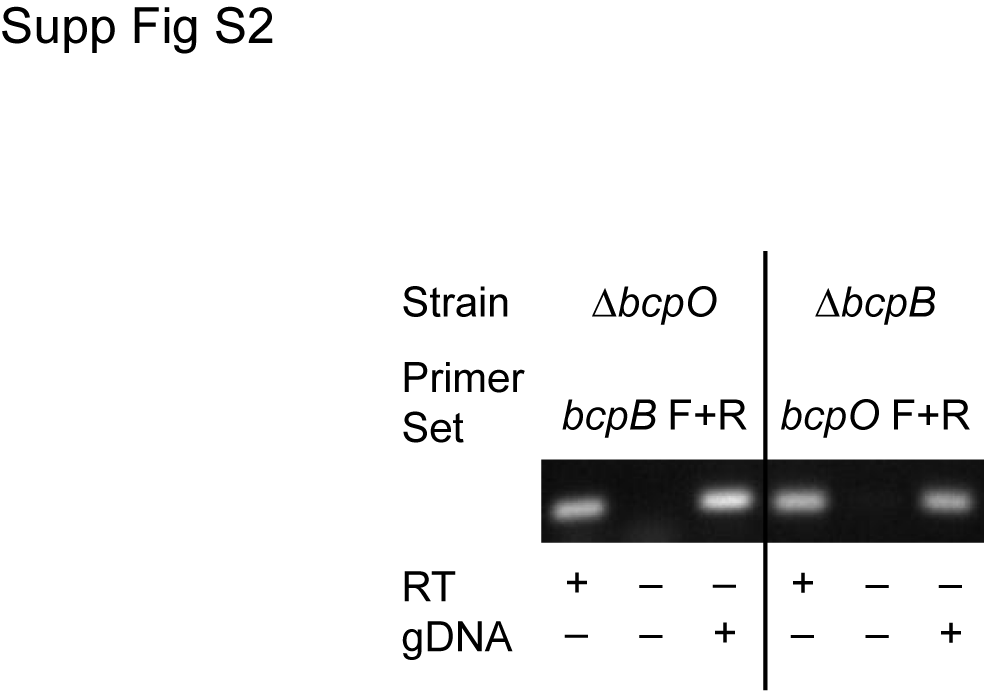

Supplement: Figure S2 — Evidence for lack of transcription polar effects in ΔbcpO and ΔbcpB strains. RT-PCR was performed on RNA extracted from ΔbcpO and ΔbcpB strains with primer sets annealing internal to bcpB (bcpB F+R) and bcpO (bcpO F+R), respectively. PCR was also performed on genomic E264 DNA as a control. (TIF) [file pgen.1002877.s002.tif]

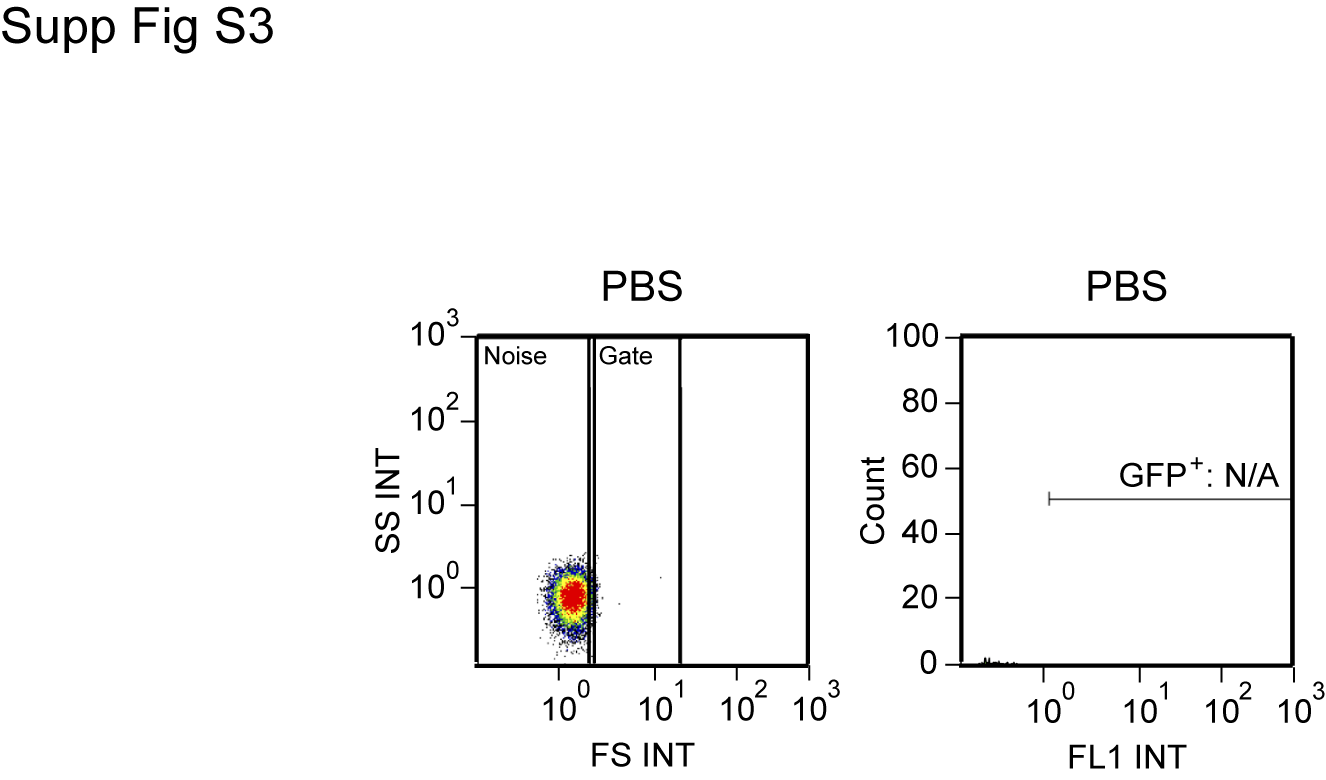

Supplement: Figure S3 — PBS control for flow cytometry analysis. Events collected in the PBS control for flow cytometry experiments were considered background (“Noise”) and excluded from further analysis. (TIF) [file pgen.1002877.s003.tif]

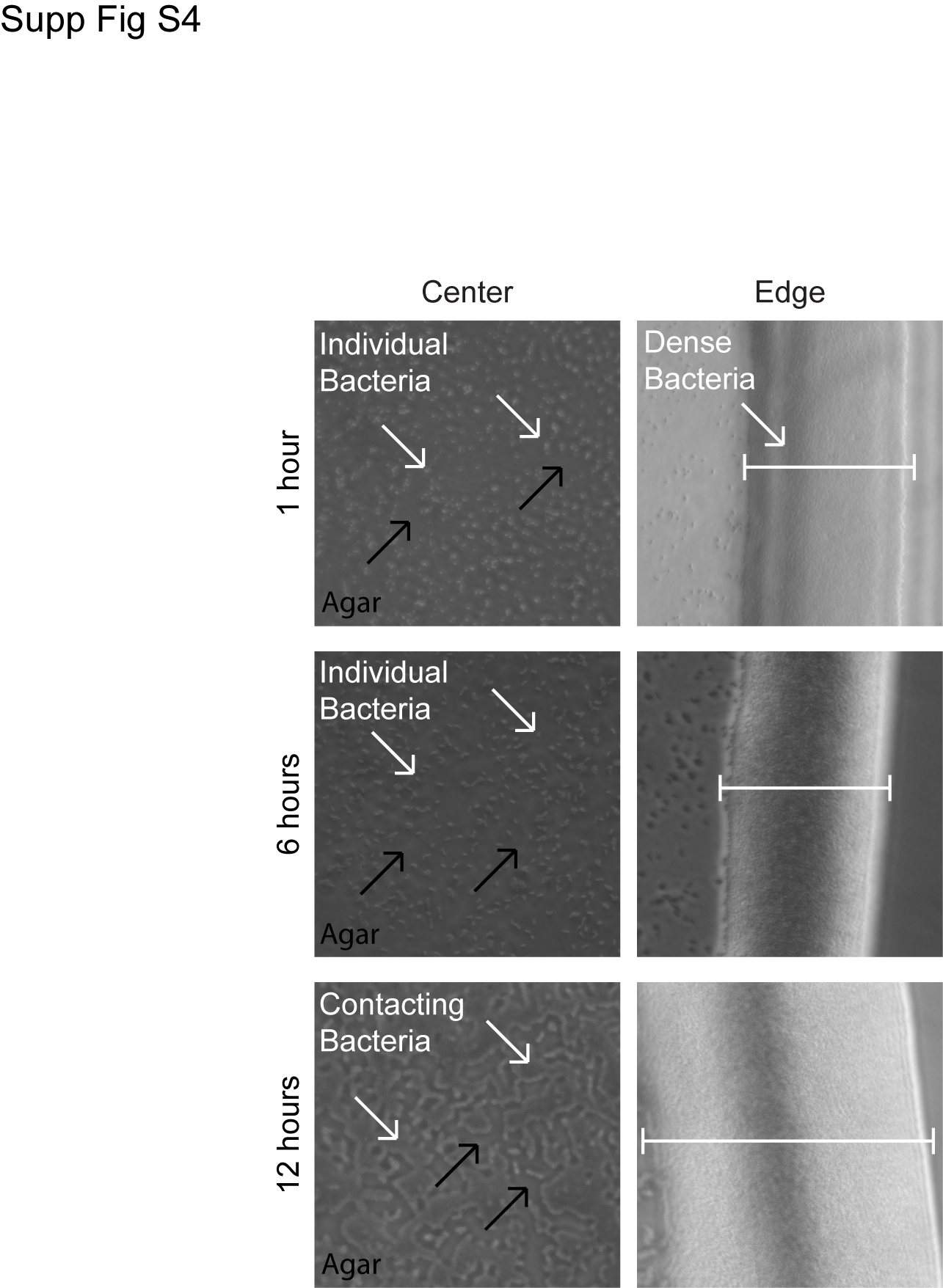

Supplement: Figure S4 — Live-image microscopy of colony biofilms on agar. Colony biofilms were imaged through LSLB agar in a glass bottom dish at 20× objective in the center and edge at the indicated times. White arrows indicate individual bacteria, contacting bacteria, or dense bacteria. Black arrows indicate agar. Images represent authentic architecture of colony biofilms. (TIF) [file pgen.1002877.s004.tif]

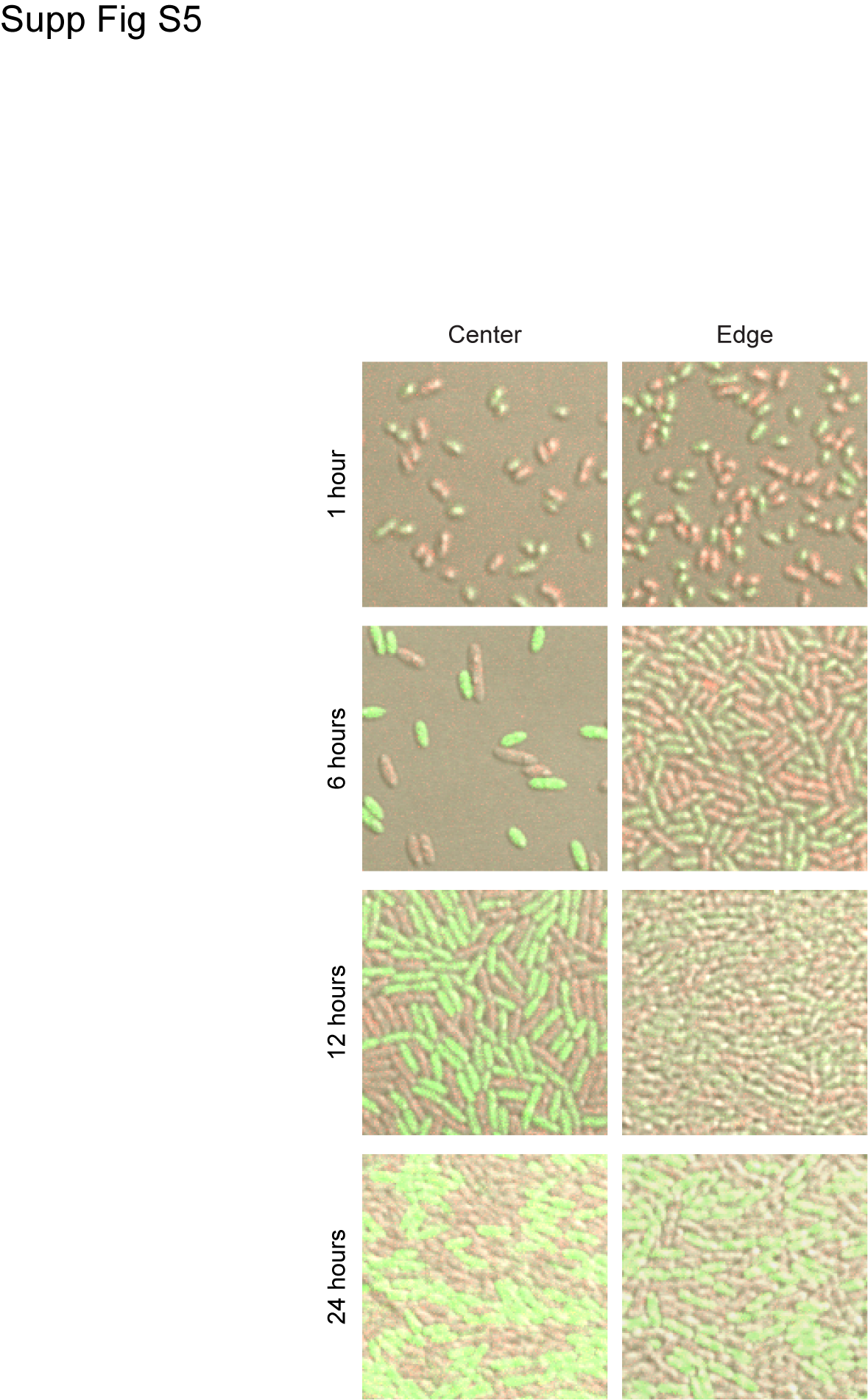

Supplement: Figure S5 — Confocal microscopy of colony biofilms on agar. Microscopy of E264PS12-rfp and E264PS12-gfp mixed at a 1∶1 ratio in the center (left column) and edge (right column) of colony biofilms at the indicated times. (TIF) [file pgen.1002877.s005.tif]
