# Supplementary material for: The Burkholderia bcpAIOB Genes Define Unique Classes of Two-Partner Secretion and Contact Dependent Growth Inhibition Systems
Source: PLoS Genet. 2012 Aug 9;8(8):e1002877. doi: 10.1371/journal.pgen.1002877 (PMC3415462; doi:10.1371/journal.pgen.1002877)
Supplement: Table S1 — Predicted Burkholderia-type CDI system-encoding loci. (PDF) [file pgen.1002877.s006.pdf]

**Supplemental Table S1** Predicted *Burkholderia*-type CDI system-encoding loci

| Locus | BcpA[1] | LYN[2] | BcpI[1] | BcpO[1] | BcpB Acc. No.[3] | Identical Alleles |
|---|---|---|---|---|---|---|
| *B. thailandensis* E264[†] | 3147 | lyn | 270 | 74 | YP_443235.1 | N/A |
| *B. thailandensis* TXDOH[†] | 3025 | lyn | 171 | 73 | ZP_02369419.1 | N/A |
| *B. pseudomallei* 1106A-1[†] | 3123 | lyn | 99 | 73 | YP_001068100.1 | *Bp*BCC215-2, *Bg*BSR3-1 |
| *B. pseudomallei* 1106A-2[†] | 3131 | lyn | 117 | 74 | YP_001065412.1 | *Bp*B7210-2, *Bp*1710b-1, *Bp*112-2, *Bp*Pasteur52237 |
| *B. pseudomallei* 1106A-3[†] | 3141 | lyn | 109 | 57* | YP_001076829.1 | *Bp*DM98, *Bp*S13, *Bp*1710b-2, *Bp*B7210-1, *Bp*1655, *Bp*112-1 |
| *B. pseudomallei* 1710b-1[†] | 3131 | lyn | 117 | 74 | YP_332689.1 | *Bp*1106A-2, *Bp*B7210-2, *Bp*112-2, *Bp*Pasteur52237 |
| *B. pseudomallei* 1710b-2[†] | 3141 | lyn | 109 | 57* | YP_336326.1 | *Bp*DM98, *Bp*S13, *Bp*1106A-3, *Bp*B7210-1, *Bp*1655, *Bp*112-1 |
| *B. pseudomallei* 668[†] | 3134 | lyn | 118 | 74 | YP_001058175.2 | N/A |
| *B. pseudomallei* K96243[†] | 3103 | lyn | 109 | 172 | YP_112054.1 | *Bp*91 |
| *B. pseudomallei* 1026b | 3122 | lyn | 101 | 81 | – | N/A |
| *B. pseudomallei* 112-1 | 3141 | lyn | 109 | 57* | ZP_02503380.1 | *Bp*DM98, *Bp*S13, *Bp*1710b-2, *Bp*1106A-3, *Bp*B7210-1, *Bp*1655 |
| *B. pseudomallei* 112-2 | 2839 | lyn | 117 | 74 | ZP_02497140.1 | *Bp*1106A-2, *Bp*B7210-2, *Bp*1710b-1, *Bp*Pasteur52237 |
| *B. pseudomallei* 14 | 3057 | lyn | 166 | – | ZP_02416760.1 | N/A |
| *B. pseudomallei* 1655 | 3141 | lyn | 109 | 57* | ZP_04891151.1 | *Bp*DM98, *Bp*S13, *Bp*1710b-2, *Bp*1106A-3, *Bp*B7210-1, *Bp*112-1 |
| *B. pseudomallei* 305 | 3107 | lyn | 96 | 74 | ZP_01767849.1 | *Bp*MSHR346, *Bp*7894-1, *Bp*406e, *Bp*576-2 |
| *B. pseudomallei* 406e | 3107 | lyn | 96 | 74 | ZP_04965785.1 | *Bp*MSHR346, *Bp*305, *Bp*7894-1, *Bp*576-2 |
| *B. pseudomallei* 576-1 | 3083 | lyn | 99 | 81 | ZP_03453873.1 | N/A |
| *B. pseudomallei* 576-2 | 3107 | lyn | 96 | 74 | ZP_03451717.1 | *Bp*MSHR346, *Bp*305, *Bp*7894-1, *Bp*406e |
| *B. pseudomallei* 7894-1 | 2932 | lyn | 96 | 74 | ZP_02487784.1 | *Bp*MSHR346, *Bp*305, *Bp*406e, *Bp*576-2 |
| *B. pseudomallei* 7894-2 | 3125 | lyn | 88 | 73 | ZP_02483696.1 | N/A |
| *B. pseudomallei* 7894-3 | 2980 | lyn | 83 | – | ZP_02487474.1 | N/A |
| *B. pseudomallei* 91 | 3103 | lyn | 109 | 172 | ZP_02452844.1 | *Bp*K96243 |
| *B. pseudomallei* B7210-1 | 3141 | lyn | 109 | 57* | | *Bp*DM98, *Bp*S13, *Bp*1710b-2, *Bp*1106A-3, *Bp*1655, *Bp*112-1 |
| *B. pseudomallei* B7210-2 | 2956 | lyn | 117 | 74 | ZP_02470328.1 | *Bp*1106A-2, *Bp*1710b-1, *Bp*112-2, *Bp*Pasteur52237 |
| *B. pseudomallei* B7210-3 | 3223 | lyn | 97 | – | ZP_02477263.1 | N/A |
| *B. pseudomallei* BCC215-1 | 3122 | lyn | 101 | 147 | ZP_02511212.1 | N/A |
| *B. pseudomallei* BCC215-2 | 3123 | lyn | 99 | 73 | ZP_02511816.1 | *Bp*1106A-1, *Bg*BSR3-1 |
| *B. pseudomallei* DM98 | 3141 | lyn | 109 | 57* | ZP_02408285.1 | *Bp*1106A-3, *Bp*S13, *Bp*1710b-2, *Bp*B7210-1, *Bp*1655, *Bp*112-1 |
| *B. pseudomallei* MSHR346 | 3107 | lyn | 96 | 74 | YP_002895846.1 | *Bp*305, *Bp*7894-1, *Bp*406e, *Bp*576-2 |
| *B. pseudomallei* NCTC13177 | 3096 | lyn | 111 | 81 | ZP_02495130.1 | N/A |
| *B. pseudomallei* Pasteur52237 | 3131 | lyn | 117 | 74 | ZP_04897242.1 | *Bp*1106A-2, *Bp*B7210-2, *Bp*1710b-1, *Bp*112-2 |
| *B. pseudomallei* S13 | 2850 | lyn | 109 | 57* | NZ_CH899763.1 | *Bp*DM98, *Bp*1710b-2, *Bp*1106A-3, *Bp*B7210-1, *Bp*1655, *Bp*112-1 |

| | | | | | | |
|---|---|---|---|---|---|---|
| *B. ambifaria* AMMD[†] | 3028 | lyn | 155 | 89 | YP_775670.1 | N/A |
| *B. ambifaria* IOP40-10 | 3066 | lyn | 89** | – | ZP_02889733.1 | N/A |
| *B. ambifaria* MEX-5 | 3776 | lyn | 128 | 99 | ZP_02907827.1 | N/A |
| *B. cenocepacia* H111 | 3229 | – | 142 | 59 | CCE53449.1 | N/A |
| *B. dolosa* AUO158-1 | 3379 | lyn | 270 | 89** | ZP_04945975.1 | N/A |
| *B. dolosa* AUO158-2 | 3037 | lyn | 164 | 67** | ZP_04947043.1 | N/A |
| *B. gladioli* BSR3-1 | 3123 | lyn | 99 | 73 | YP_004358894.1 | *Bp*1106A-1, *Bp*BCC215-2 |
| *B. gladioli* BSR3-2(p) | 3082 | lyn | 103 | – | YP_004350998.1 | N/A |
| *B. gladioli* BSR3-3 | 3038 | lyn | 77 | 113 | YP_004349107.1 | N/A |
| *B. glumea* BGR1[†] | 3151 | lyn | 269 | 71 | YP_002909358.1 | N/A |
| *B. multivorans* CGD2M-1 | 3060 | lyn | 93 | 118 | ZP_03577349.1 | N/A |
| *B. multivorans* CGD2M-2 | 3423 | venn | 106 | – | ZP_03575068.1 | N/A |
| *B. phymatum* STM815-1[†] | 3079 | – | 44 | – | YP_001860455.1 | N/A |
| *B. phymatum* STM815-2(p)[†] | 3015 | – | 187 | 54 | YP_001863347.1 | N/A |
| *B. rhizoxinica* HKI454[†] | 2981 | – | 83 | – | YP_004028152.1 | N/A |
| *B. ubonensis* BU | 2814 | lyn | 89 | 56 | ZP_02379486.1 | N/A |
| *B. vietnamiensis* G4[†] | 3513 | lyn | 81 | 64 | YP_001116443.1 | N/A |
| *B. xenovorans* LB400[†] | 2984 | – | 81 | 83 | YP_557396.1 | N/A |
| *R. solanacearum* CFBP2957(p)[†] | 3651 | – | 301 | 143 | YP_003747808.1 | N/A |
| *R. solanacearum* CMR15(p)[†] | 3552 | – | 145 | 59 | CBJ40172 | N/A |
| *R. solanacearum* GMI1000[†] | 3501 | lyn | 80 | 155 | NP_519009.1 | N/A |
| *R. solanacearum* Po82-1[†] | 3305 | venn | 182* | 179 | AEG67591.1 | N/A |
| *R. solanacearum* Po82-2(p)[†] | 3177 | – | 190 | 76 | AEG71437.1 | N/A |
| *R. solanacearum* PSI07[†] | 3386 | – | 166 | 51 | YP_003750973.1 | N/A |
| *R. syzygii* R24 | 3208 | – | 186 | 86 | CCA87914.1 | N/A |
| *C. metallidurans* CH34(p)[†] | 3004 | – | 77 | 63 | YP_587468.1 | N/A |

[1]Amino acid length of predicted protein

[2]Nx(E/Q)LYN (or VENN) motif in predicted BcpA sequence located ~350 amino acid from the C-terminus of the protein

[3]Accession number in NCBI database

[†]Loci from complete genome sequences

*Encoding ORF overlaps the 3' ORF by ~50-100 nucleotides

**Encoding ORF begins with a 'gtg' sequence

(p) Locus located on plasmid

N/A Allele not found in any other strain
