# Supplementary material for: The Burkholderia bcpAIOB Genes Define Unique Classes of Two-Partner Secretion and Contact Dependent Growth Inhibition Systems
Source: PLoS Genet. 2012 Aug 9;8(8):e1002877. doi: 10.1371/journal.pgen.1002877 (PMC3415462; doi:10.1371/journal.pgen.1002877)
Supplement: Table S2 — Quantification of bcpA-gfp expression by flow cytometry. (PDF) [file pgen.1002877.s007.pdf]

Supplemental Table S2 Quantification of *bcpA-gfp* expression by flow cytometry

**Expt. No.**	**Medium**	**Strain**	**Gated Events**	**GPF⁺ Events**	**% GFP⁺**	**Fluorescence Intensity***
1	LSLB	P_{bcpA}-*gfp*	182,217	79	0.04	2.75
2	LSLB	P_{bcpA}-*gfp*	182,302	800	0.44	2.93
3	LSLB	P_{bcpA}-*gfp*	238,236	914	0.38	2.64
Mean ± SD	LSLB	P_{bcpA}-*gfp*	–	–	**0.29 ± 0.22**	**2.77 ± 0.15**
1	LSLB	P_{S12}-*gfp*	194,917	193,863	99.46	3.11
2	LSLB	P_{S12}-*gfp*	188,610	187,252	99.28	3.14
3	LSLB	P_{S12}-*gfp*	219,682	217,706	99.10	2.86
Mean ± SD	LSLB	P_{S12}-*gfp*	–	–	**99.28 ± 0.18**	**3.04 ± 0.15**
1	LSLB	vector	189,720	9	0.00	2.41
2	LSLB	vector	177,556	26	0.01	2.30
3	LSLB	vector	228,407	6	0.00	2.56
Mean ± SD	LSLB	vector	–	–	**0.00 ± 0.01**	**2.42 ± 0.13**
1	M63	P_{bcpA}-*gfp*	216,480	514	0.24	2.81
2	M63	P_{bcpA}-*gfp*	249,142	423	0.17	3.24
3	M63	P_{bcpA}-*gfp*	150,310	194	0.13	2.45
Mean ± SD	M63	P_{bcpA}-*gfp*	–	–	**0.18 ± 0.06**	**2.83 ± 0.40**
1	M63	P_{S12}-*gfp*	230,161	228,164	99.13	3.13
2	M63	P_{S12}-*gfp*	123,061	122,264	99.35	3.50
3	M63	P_{S12}-*gfp*	175,807	169,239	96.26	2.20
Mean ± SD	M63	P_{S12}-*gfp*	–	–	**98.25 ± 1.72**	**2.94 ± 0.67**
1	M63	vector	135,0.26	1	0.00	1.48
2	M63	vector	100,010	0	N/A	N/A
3	M63	vector	160,154	110	0.07	1.98
Mean ± SD	M63	vector	–	–	**0.02 ± 0.04**	**1.73 ± 0.35**

*Mean relative fluorescence intensity of gated GFP⁺ events
