# Supplementary material for: The Burkholderia bcpAIOB Genes Define Unique Classes of Two-Partner Secretion and Contact Dependent Growth Inhibition Systems
Source: PLoS Genet. 2012 Aug 9;8(8):e1002877. doi: 10.1371/journal.pgen.1002877 (PMC3415462; doi:10.1371/journal.pgen.1002877)
Supplement: Table S3 — RT-PCR primer sequences. (PDF) [file pgen.1002877.s008.pdf]

Supplemental Table S3 RT-PCR primer sequences

Primer Set	Forward Primer[1]	Reverse Primer[1]
1F, 1R	ACTGGACCGACATGCTCGAG	ATAGCCTGTCGATCGGCTTTGGTT
2F, 2R	TCAACGGCAGCCTTCCAATACAGA	GGCAACACGGGCTCCATTTCTG
3F, 3R	GTTTGGCATGTCGGCGCTTCTC	GTCGATGCCGAGCGACAGATTG
50ntF, *bcpA*R	CCGTGAGGGCGGATCGAACG	TGCCACCTAACTGAGCTACGTCAA
70ntF, *bcpA*R	ATTAGATTCCATGCGATGTTCGAGG	TGCCACCTAACTGAGCTACGTCAA
120ntF, *bcpA*R	ATCTCGGCAACGATTGACACTCT	TGCCACCTAACTGAGCTACGTCAA
150ntF, *bcpA*R	GAGCAATGGAGTTGTTTCGTCCGTC	TGCCACCTAACTGAGCTACGTCAA
200ntF, *bcpA*R	GAGTATGTCGTTATCCAAAATATCGAGATAG	TGCCACCTAACTGAGCTACGTCAA
250ntF, *bcpA*R	GTAAGATTCGCGCGGATTGCGATTAA	TGCCACCTAACTGAGCTACGTCAA
300ntF, *bcpA*R	CGACCTCGCAATTCTGTCAGGGATG	TGCCACCTAACTGAGCTACGTCAA
*bcpB*F, *bcpB*R	CGATGTCGTACTCGACGTGAAGCG	GTCGATGCCGAGCGACAGATTG
*bcpO*F, *bcpO*R	GTTTGGCATGTCGGCGCTTCTC	GGCAACACGGGCTCCATTTCTG

[1]Primer sequences are listed 5' to 3'
